# Single-cell RNA sequencing reveals developmental trajectories and environmental regulation of callus formation in Arabidopsis

**DOI:** 10.1007/s44154-025-00255-4

**Published:** 2025-09-12

**Authors:** Zhixin Liu, Yixin Zhang, Qianli Zhao, Hao Liu, Yaping Zhou, Aizhi Qin, Chunyang Li, Lulu Yan, Mengfan Li, Peibo Gao, Xiao Song, Yajie Xie, Enzhi Guo, Luyao Kong, Liping Guan, Guoyong An, Xuwu Sun

**Affiliations:** National Key Laboratory of Cotton Bio-Breeding and Integrated Utilization, State Key Laboratory of Crop Stress Adaptation and Improvement, School of Life Sciences, Henan University, Kaifeng, 475001 China

**Keywords:** Plant regeneration, Callus cells, Dedifferentiation, ScRNA-seq, Developmental trajectories, Environmental factors

## Abstract

Plant cells exhibit an extraordinary regenerative potential, achieving cellular totipotency by dedifferentiating to form new tissues. While significant progress has been made in understanding cell fate mechanisms, the regulatory networks governing callus cell development remain insufficiently explored, particularly regarding cell classification, morphology, and regulatory processes. This study provides a detailed investigation into the developmental dynamics and transcriptomic profiles of callus cells in Arabidopsis at key stages: initiation, proliferation, and greening. Employing single-cell RNA sequencing and UMAP-based clustering, we annotated cell clusters based on highly enriched gene expressions. Developmental trajectories were further mapped through pseudotime analysis, revealing distinct transcription factor networks. Additionally, functional analysis of key regulatory genes was conducted using mutant and overexpression lines, affirming their roles in callus development. Gene Ontology analysis highlighted the involvement of environmental factors—low oxygen and salinity promoted callus formation, while light inhibited it, though essential for greening. These findings shed light on the complex regulatory landscape of plant tissue regeneration and guide future research avenues.

**Supplementary Information:**

The online version contains supplementary material available at 10.1007/s44154-025-00255-4.

## Introduction

Callus formation is a critical step in plant regeneration and is categorized into embryogenic and non-embryogenic callus, each derived from distinct regeneration pathways. Embryogenic callus is comparable to pluripotent stem cells, while non-embryogenic callus resembles multipotent stem cells (Atta et al. 2009). Research indicates that the formation of non-embryogenic callus, regardless of the explant’s origin, occurs through a lateral root initiation program (Xu 2018). When explants are detached from the plant, the cells typically enter a dormant, non-dividing state due to the presence of endogenous inhibitors of cell division. However, external stimuli, such as plant hormones, can lift this inhibition, allowing differentiated dormant cells to regain their capacity for division (Grafi and Barak 2015).

Before cell division occurs, the wounding of the explant triggers rapid signaling that promotes the synthesis of endogenous auxin and its accumulation at the cut surfaces of cells with regenerative potential (Duclercq et al. 2011). Auxin signaling activates the expression of *WUSCHEL RELATED HOMEOBOX 11* (*WOX11*) and *WOX12* transcription factors, which direct the transformation of regenerative cells into root progenitor cells (Motte et al. 2014; Hellmann and Helariutta 2019). These transcription factors serve as markers of root progenitor cells and regulate their development. Following this, root progenitor cells undergo division, leading to the formation of root primordium cells. Throughout this process, the number of cells increases significantly, tissue dry weight grows, and the structure becomes more loosely organized, forming callus characterized by *WOX5* expression (Che et al. 2007). Toward the end of callus formation, some cells may begin to differentiate due to uneven contact with the culture medium or a decrease in external hormone levels. Subsequently, root primordium cells continue to divide and differentiate into either the root apical meristem (RAM) or shoot apical meristem (SAM) (Xu 2018). Rosspopoff et al. (2017) investigated the direct conversion of LRP into SM and found that, following cytokinin treatment, root primordia could transition into actively growing shoots without the intermediate formation of proliferative callus. This transformation is confined to a narrow temporal window during the early stages of LRP development and necessitates the presence of apical stem cells. Remarkably, within this critical period, the organogenesis programs of roots and shoots exhibit substantial plasticity, allowing for repeated and reversible shifts in the stem cell niche state (Rosspopoff et al. 2017).

In vitro, callus formation is induced by the addition of auxin and cytokinin. Auxin-induced callus formation occurs through two main pathways. In the first pathway, auxin-responsive transcription factors AUXIN RESPONSE FACTOR 7 (ARF7) and ARF19 regulate the expression of LBD family transcription factors, Such as LATERAL ORGAN BOUNDARIES-DOMAIN 16 (LBD16), LBD17, LBD18, and LBD29 (Fan et al. 2012; Lee et al. 2009). Studies have also shown that AtbZIP59 forms a protein complex with LBDs to facilitate callus induction (Xu et al. 2018). The second pathway involves auxin signaling through the PRZ1/AtADA2 protein, which mediates the inhibition of cyclin-dependent kinase inhibitors, Such as KIP-RELATED PROTEIN 2 (KRP2), KRP3, and KRP7 (Anzola et al. 2010). Similarly, cytokinin-induced callus development is regulated by two distinct pathways. In the first pathway, cytokinin signaling depends on Type-B ARR transcription factors, which regulate the expression of the *CYCLIN D3;1* (*CYCD3;1*) cyclin (Dewitte et al. 2007). The second pathway involves the induction of AP2/ERF family transcription factors, such as ESR1, which reactivates the cell cycle by promoting the expression of *CYCD1;1* and *OBF BINDING PROTEIN 1* (*OBP1*) (Ikeda et al. 2006). The AP2 family transcription factor PUCHI also plays a role in callus formation (Trinh et al. 2019). Wound-induced callus formation is primarily driven by the upregulation of wound-induced genes such as *WOUND INDUCED DEDIFFERENTIATION 1* (*WIND1*), *WIND2*, *WIND3*, and *WIND4*, which promote cytokinin responses and subsequently facilitate callus formation (Iwase et al. 2017, 2021). Furthermore, callus can also be induced by regaining embryonic or meristematic fates (Chen et al. 2019; Boutilier et al. 2002).

Greening and bud formation in callus are critical indicators of its redifferentiation potential. Callus greening serves as a reliable marker for its capacity for bud formation. Upon transfer to shoot induction medium (SIM) and exposure to standard light conditions, the tumorous tissue at the callus-medium interface transitions from yellowish-white to green, with green bud primordia becoming visible after several days (Che et al. 2007). Over time, the number of these bud primordia increases. The regenerated buds arise either directly or indirectly from cycling cells adjacent to the xylem poles (Atta et al. 2009). Numerous studies have identified WUSCHEL (WUS) as a key regulator of bud regeneration (Motte et al. 2014; Leibfried et al. 2005). WUS not only marks shoot stem cells but also identifies the SAM, analogous to the role of WOX5 in root stem cells. Following several days of incubation in SIM, the callus begins to express WUS in specific regions, giving rise to shoot progenitor cells that subsequently differentiate into SAM. The maintenance of stem cell identity within SAM is tightly regulated by WUS, which promotes the expression of CLAVATA3 (CLV3). CLV3 encodes a small peptide that binds to the receptor-like kinase CLAVATA1 (CLV1), inhibiting *WUS* expression and thereby maintaining stem cell homeostasis. As the stem cell population declines, CLV3 levels decrease, allowing *WUS* expression to rise and thus expanding the stem cell population (Muller et al. 2008; Cheng et al. 2010).

Furthermore, cytokinin regulates WUS expression through two distinct pathways: the CK-ARRs pathway and the CK-CYCD3-E2FA-MET1 pathway (Xie et al. 2018). In the former pathway, histone H3 lysine 27 trimethylation (H3K27me3) is removed from the WUS locus in a cell cycle-dependent manner, while Type-B ARR transcription factors activate WUS by binding to its promoter region, in conjunction with HD-ZIP III transcription factors (Dewitte et al. 2007; Liu et al. 2020a). In the latter pathway, MET1 initially represses *WUS* expression in the callus (Shemer et al. 2015). Following SIM-mediated bud induction, MET1 relocates to the uppermost layer, while *WUS* is expressed just beneath it. Cytokinin plays a crucial role in bud formation, with the auxin-to-cytokinin ratio influencing SAM differentiation (Su et al. 2011). There is an antagonistic relationship between the auxin biosynthesis gene YUCCA and Type-B ARRs, while the interaction between ARFs and MET1 promotes the mutual exclusion of auxin and cytokinin signaling, thereby driving WUS expression in cytokinin-rich regions (Xie et al. 2018; Ikeuchi et al. 2013).

MicroRNAs (miRNAs), which are small RNAs ranging from 21 to 24 nucleotides in length, function as the final products of non-coding RNA genes and play crucial roles in plant development and signal transduction. The auxin receptor TIR1 promotes bud regeneration by upregulating *WUS* and *CLV3*, whereas miR393 suppresses bud regeneration by inhibiting *TRANSPORT INHIBITOR RESPONSE 1* (*TIR1*) (Chen 2005; Chen et al. 2011). miR164 downregulates the expression of NAC family members *CUC1* and *CUC2* (Chen 2005), which serve as marker genes for SAM. Double mutants of *cuc1 cuc2* fail to form SAM during normal development (Hibara et al. 2003; Motte et al. 2011). miR156 targets the SPL transcription factor family, which is involved in plant phase transitions and flowering. SPL9 interacts with Type-B ARR to reduce plant sensitivity to cytokinin, resulting in a decline in bud regeneration capacity (Wu et al. 2009). miR160 regulates the expression of *WUS* and *CLV3* by cleaving its target gene, *ARF10*, leading to its degradation (Qiao and Xiang 2013). The transcription factor ARF5 (also known as MP) not only influences auxin signaling but also promotes bud formation in *Arabidopsis thaliana* (Arabidopsis) callus by regulating the cytokinin pathway in shoot branching (Ckurshumova et al. 2014). Moreover, ARF5 has been shown to regulate *miR390* expression in meristems, playing a role in auxin signaling cascades to ensure signal sensitivity and stability (Dastidar et al. 2019).

The formation of callus is influenced by a range of factors that can be broadly categorized into internal and external determinants. Internal factors primarily encompass the plant’s genetic characteristics and physiological state. Even within the same plant, the morphogenic capacity of various tissues can be influenced by factors such as the plant’s age, seasonal changes, and physiological conditions, leading to variations in callus formation efficiency. Sterile seedlings with leaf widths of approximately 0.5 cm are ideal for sampling; if sampled too early, the leaves may be underdeveloped, yielding insufficient explants, whereas sampling too late may increase the risk of dedifferentiation.

External factors encompass components of the culture medium—such as plant hormones, inorganic salts, and organic nutrients—as well as environmental conditions like temperature, humidity, and light in the culture room. Gene expression is affected by the physical and chemical conditions present during tissue culture. Among these factors, the influence of plant hormones on callus formation has become a significant focus of research (Jiménez 2005). Studies have shown that a higher auxin-to-cytokinin ratio favors callus and adventitious root formation, whereas a lower ratio promotes bud formation (Skoog and Miller 1957). Additionally, the selection of carbon source significantly affects callus induction rates, with sucrose-containing media yielding higher induction rates due to sucrose’s superior utilization efficiency (Fellers et al. 1995).

The process of shoot induction from callus involves a gradual decrease in the ratio of endogenous auxins to cytokinins. When callus is transferred to shoot induction medium (SIM), its differentiation may be slow, potentially resulting in browning or death, which can be attributed to insufficient auxin levels or inappropriate temperature. Conversely, excessively high auxin concentrations can lead to uncontrolled cell division without differentiation, exacerbated by factors such as insufficient light or excessive heat.

Light conditions—including light intensity, photoperiod, and light quality—also influence the greening of callus. Extended photoperiods tend to enhance greening, possibly due to the positive correlation between light duration and chlorophyll accumulation within a certain range (Capite 1955). Moreover, light quality may play a role in the morphogenesis of specific organs during tissue culture (Batista et al. 2018).

Our understanding of the regulatory mechanisms governing the developmental processes of callus cells during regeneration remains limited, particularly regarding cell types, classification, morphological features, and their regulatory mechanisms. A detailed exploration of the cellular morphology, classification, developmental dynamics, and transcriptomics of callus cells is essential. Single-cell RNA sequencing (scRNA-seq) is a novel technology that enables the amplification and sequencing of the entire transcriptome at the single-cell level. This method can reveal cellular heterogeneity, identify distinct cell types, and infer the temporal and spatial trajectories of cell development (Tang et al. 2009). Owing to its significant advantages, scRNA-seq has been widely applied in animal and human medical research to study the cell types and developmental dynamics of immune cells, tumor cells, and stem cells. It has facilitated the generation of developmental maps and gene expression profiles in human heart, lung, bone marrow, and thymic cells, thus aiding advancements in medical research (Asp et al. 2019; Angelidis et al. 2019; Baccin et al. 2020; Park et al. 2020).

In plants, the application of scRNA-seq has been relatively delayed due to the challenges associated with dissociating cells caused by the presence of the plant cell wall. In 2016, Efroni et al. employed scRNA-seq to investigate root tip regeneration in Arabidopsis following root tip excision, establishing a model for stem cell regeneration and demonstrating the involvement of multiple cell types in stem cell reconstitution (Efroni et al. 2016). Subsequently, Ryu et al. generated a gene expression atlas of Arabidopsis root cells at the single-cell level (Ryu et al. 2019). In the same year, Denyer et al. constructed a fine-scale gene regulatory network for Arabidopsis root cells, identifying key regulators of root cell differentiation and their downstream targets (Denyer et al. 2019). Liu et al. elucidated the developmental process of Arabidopsis leaf stomatal cells, revealing that BPC and WRKY33 are critical transcription factors involved in stomatal development (Liu et al. 2020b). Kim et al. examined Arabidopsis leaf vein vascular cells, demonstrating that phloem parenchyma (PP) development does not rely on APL, a transcription factor essential for phloem differentiation, and suggesting that PP plays roles in sugar and amino acid transport (Kim et al. 2021). Zhai and Xu (2021) utilized scRNA-seq to uncover mechanisms of pluripotency acquisition during callus formation, linking the intermediate cell layer to stem cell-like transcriptional activity in organ regeneration (Zhai and Xu 2021).

Despite these advancements, the regulatory mechanisms involved in callus initiation, proliferation, and greening remain unclear. This study leverages scRNA-seq technology to analyze callus cells during the initiation, proliferation, and greening phases in Arabidopsis. We identified distinct cell types and the genes specifically expressed in these cell types. Through transcriptional regulatory network analysis, we identified key genes and signaling pathways involved in callus development and proliferation.

## Results

### Dynamic morphological observation of callus development in Arabidopsis leaf and root tissues

**Fig. 1:**
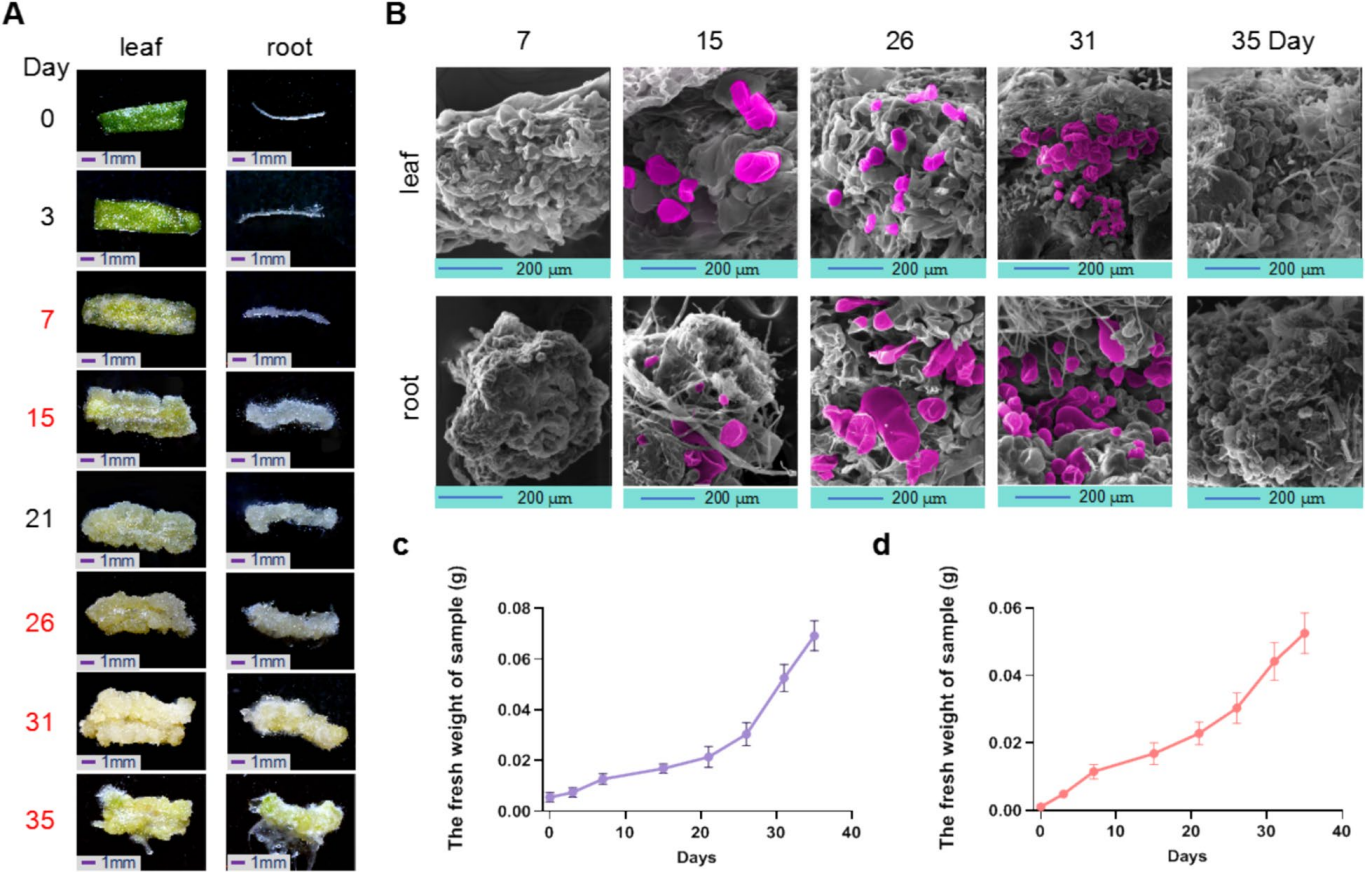
Dynamic analysis of callus development in Arabidopsis leaves and roots. **A** The comprehensive phenotypic observation was conducted to analyze the induction and developmental dynamics of callus tissues in both leaf and root organs. The callus induction phase spanned from day 0 to day 7, followed by a proliferation phase from day 15 to day 26. From day 31 to day 35, callus tissues were transferred to light conditions for continued growth and greening, exhibiting distinct developmental morphologies. **B** Scanning electron microscopy (SEM) images illustrate the structural characteristics and cellular morphology of leaf and root calli at various developmental stages. **C** Fresh weight analysis of leaf-derived calli. **D** Fresh weight analysis of root-derived calli. For both (**C**) and (**D**), fresh weight measurements were performed by weighing ten calli at each time point, and the average weight per callus was calculated. The experiment was repeated three times

To investigate the initiation and molecular regulatory mechanisms underlying callus formation in wild type (WT) Arabidopsis leaf and root tissues, we independently cultured leaf and root explants on induction medium. The developmental progression of callus formation was monitored over time, and phenotypic changes were documented (Fig. 1A). After 7 days of culture, callus cells—characterized by their loose, irregular arrangement of thin-walled, light, and transparent cells—were first observed in both root and leaf tissues. By day 15, a pronounced increase in callus proliferation was evident, particularly in the root explants, where extensive callus growth covered the entire root Surface. After 26 days, further thickening of the callus was observed in leaf tissues, which adopted a pale yellow hue, while root callus exhibited similar thickening and gradual lightening. By day 31, callus from both tissue types displayed comparable morphological and chromatic characteristics. After 35 days of cultivation under light conditions, some callus cells began to green, entering a pre-budding phase, during which structures resembling trichomes and root hairs became apparent on the surface.

To further characterize cellular development within the callus, scanning electron microscopy (SEM) was employed at various stages (Fig. 1B). After 7 days, bubble-like cells formed rapidly on the Surface of the leaf callus, effectively covering the epidermis. In the root callus, newly formed protruding cells began to emerge. By day 15, an increase in the size of bubble-like cells was evident in both leaf and root calli. By day 26, the calli from leaf and root tissues exhibited morphological convergence, displaying abundant bubble-like cells in both types. By day 31, the larger bubble-like cells were gradually replaced by smaller cells, accompanied by a significant transformation in cellular morphology. In the leaf callus, diverse cell morphologies emerged, some resembling collapsed bubble-like structures, while root callus cells displayed features akin to root hairs.

By day 35, the morphology and cellular composition of callus cells in both leaf and root tissues exhibited converging characteristics. At this stage, most cells in both callus types were relatively small, resulting in a more compact arrangement. Notably, root hair-like structures also appeared in the leaf callus. To quantify the developmental rate of callus formation, we measured the weight of callus tissues from both leaf and root explants at various time points (7, 15, 26, 31, and 35 day). The data indicated a steady increase in callus weight over the first 21 days, followed by a nearly threefold increase between days 21 and 35.

### Single-cell transcriptomic analysis of callus development in Arabidopsis leaf and root tissues

To investigate the developmental characteristics of callus formation, we performed scRNA-seq on samples representing various stages of development. Six distinct time points were selected: leaf initial callus (leaf_initial_callus), leaf callus (leaf_callus), leaf callus at the greening stage (leaf_callus_greening), root initial callus (root_initial_callus), root callus (root_callus), and root callus at the greening stage (root_callus_greening).

Following quality control screening of the raw scRNA-seq data for these six samples, we identified high-quality cells meeting the criteria as follows: 18,789, 14,932, 18,779, 14,268, 10,971, and 10,695, respectively. Further quality control (QC) was conducted to eliminate low-quality cells, ensuring that the number of detected genes and unique molecular identifiers (UMIs) fell within the mean ± two standard deviations while maintaining the proportion of mitochondrial genes below 10%. After QC processing, the final cell counts for each sample were 17,379, 13,421, 17,717, 13,674, 10,213, and 9,757, respectively (Fig. S1).

### Classification and annotation of callus cells at different developmental stages in Arabidopsis leaves and roots

**Fig. 2:**
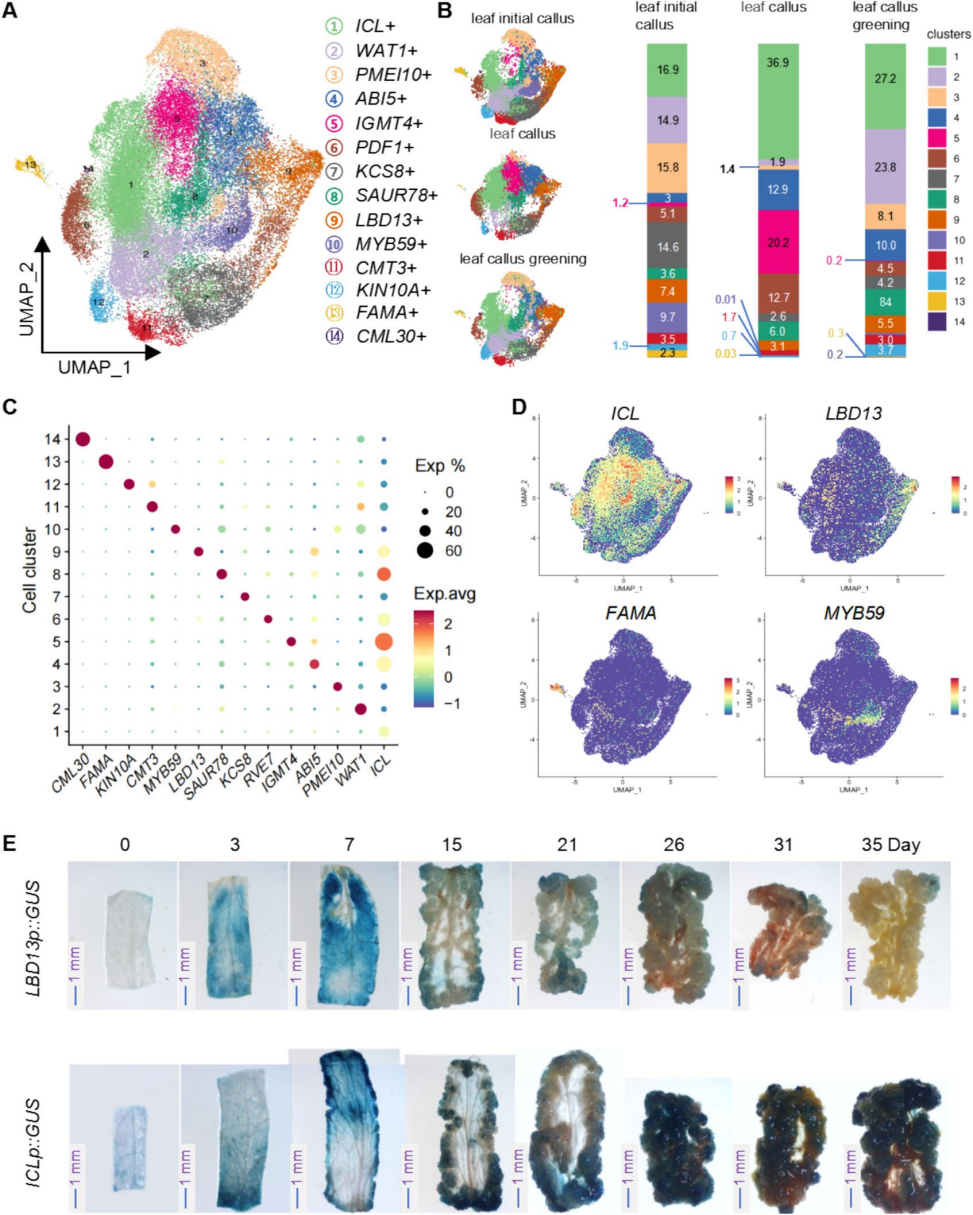
Single-cell RNA sequencing (scRNA-seq) and cell type characterization in leaf-derived callus. **A** Uniform Manifold Approximation and Projection (UMAP) projection of single-cell transcriptomic data from leaf-derived callus reveals 14 distinct cell clusters. Each cluster is defined by a unique set of marker genes, which correspond to specific cell types. **B** Quantitative analysis of the relative abundance of different cell types at various stages of callus development. Left Panel: The distribution of cells from three distinct developmental stages in the UMAP space is shown. Right Panel: The stacked bar chart illustrates the proportional representation of cells from different clusters at each developmental stage, with the numbers corresponding to the percentage of total cells within each sample. **C** Dot plot illustrating the expression levels of key marker genes across the identified cell clusters and their respective cell types. **D** Feature plots visualizing the spatial distribution and expression patterns of key marker genes within selected cell types. **E** Temporal expression patterns of representative marker genes during callus development, as analyzed using *LBD13P::GUS* and *ICLp::GUS* reporter lines, providing insights into the dynamic regulatory landscape of callus formation

Subsequently, we performed Uniform Manifold Approximation and Projection (UMAP) dimensionality reduction analysis on the filtered cells. For leaf-derived callus, the cells were categorized into 14 distinct clusters (Fig. 2A). We also screened the differentially expressed genes (DEGs) in each of clusters in leaf-derived callus samples (Table S1) and performed Gene Ontology (GO) and Kyoto Encyclopedia of Genes and Genomes (KEGG) analyses on these DEGs (Fig. S2A and Table S2). The results of the GO analysis reveal that the differentially expressed genes in distinct cell clusters are significantly enriched in several key GO terms (Fig. S2A). For instance, genes in cluster 3 and cluster 5 are markedly enriched in leaf development (Fig. S2A). Moreover, cluster 3, 4, 5, 9, and 10 exhibit significant enrichment in response to oxidative stress (Fig. S2A). The GO analysis related to energy metabolism further highlights that cluster 1, 2, 7, and 10–13 show substantial enrichment in terms associated with the mitochondrial protein-containing complex (Fig. S2A). The cluster 12 is notably enriched in terms related to the plant-type cell wall (Fig. S2A). Additionally, the differentially expressed genes in cluster 11 and Cluster 13 are significantly enriched in the aerobic respiration pathway (Fig. S2A). Concurrently, cluster 2, 7, 11, and 12 exhibit substantial enrichment in proteasome complex and ribosome terms (Fig. S2A).

Due to the lack of comprehensive research and definitions regarding specific callus cell types, we had no predefined marker genes for these cells. Thus, we defined the identified cell types based on genes uniquely expressed in each cluster (Fig. 2A). By analyzing the proportion of cells in each cluster across different samples, we found that clusters 1, 4, 5, and 6 were more prevalent in leaf callus samples, suggesting that these clusters correspond to more mature callus cells. Further analysis revealed that the *ICL* gene was primarily expressed in leaf callus, whereas the *LBD13* gene was highly expressed during the leaf initial callus stage. To validate our findings, we generated transgenic Arabidopsis lines with GUS reporter genes driven by the native promoters of these two genes. Upon examining the GUS staining signals, we observed that the GUS signal in the *LBD13p::GUS* line was highly expressed at the early stages of leaf callus development (7 days), but gradually diminished as the callus progressed. By the greening stage of callus (35 days), only weak GUS signals were detectable in localized regions of the callus. In contrast, the GUS signal in the *ICLp::GUS* line was markedly high and specific at the initiation phase of callus formation (7 days). As the callus developed, during the rapid growth phase (15–31 days), the GUS signal remained at a relatively high level. Even at the greening stage of the callus (35 days), a Substantial expression of GUS signal persisted in the callus regions. The expression patterns of these GUS reporter genes were consistent with our scRNA-seq analysis, indicating that cluster 1 (*ICL*) represents more mature callus cells, while cluster 9 (*LBD13*) corresponds to early-stage callus cells.

**Fig. 3:**
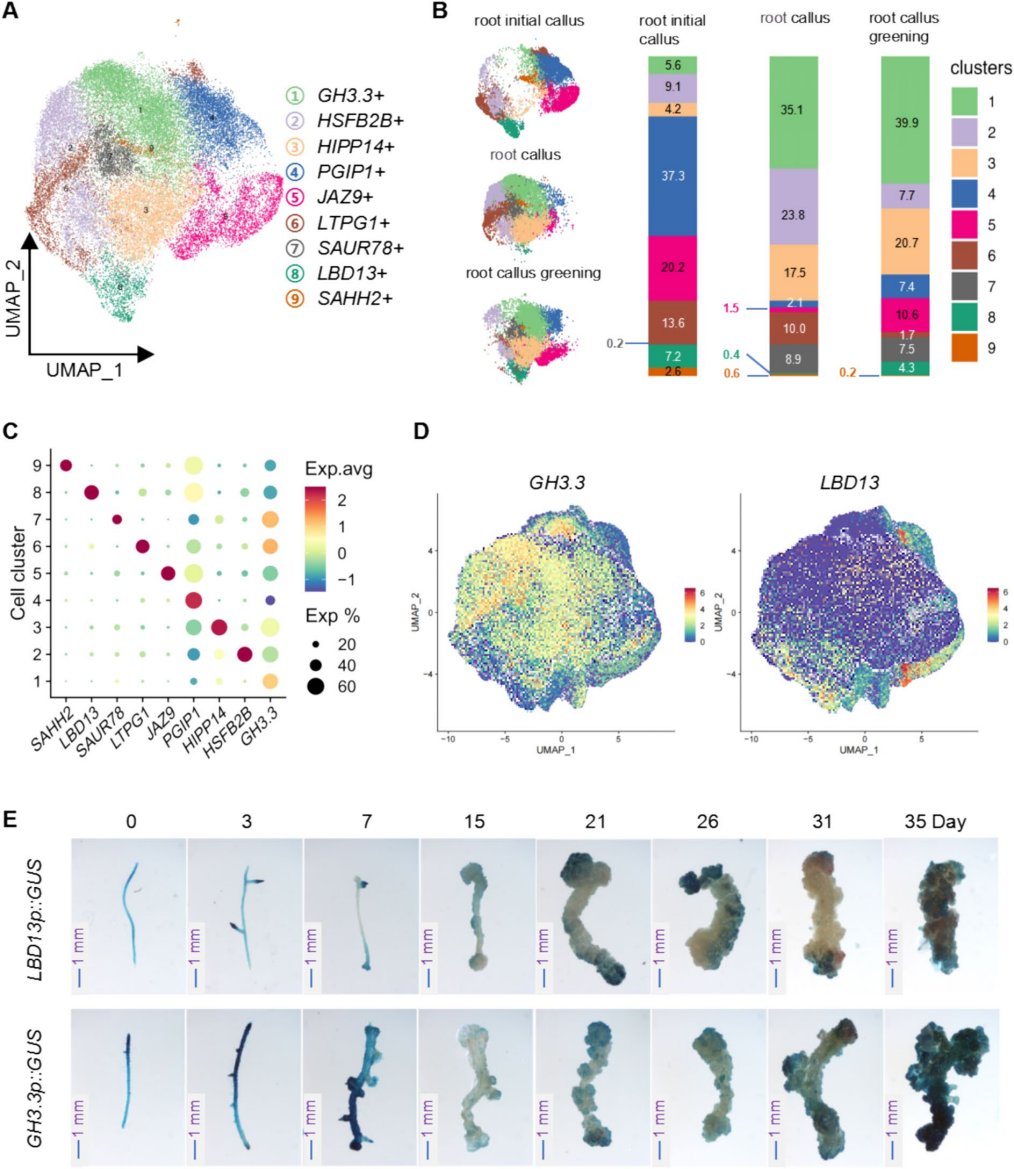
scRNA-seq analysis and cell type characterization in root-derived callus. **A** UMAP projection of single-cell transcriptomic data from root-derived callus reveals 9 distinct cell clusters, each characterized by specific marker gene expression patterns corresponding to unique cell types. **B** Quantitative assessment of the relative proportions of various cell types at different stages of root callus development. Left Panel: The distribution of cells from three distinct developmental stages in the UMAP space is shown. Right Panel: The stacked bar chart illustrates the proportional representation of cells from different clusters at each developmental stage, with the numbers corresponding to the percentage of total cells within each sample. **C** Dot plot depicting the expression levels of key marker genes across the identified cell clusters and their associated cell types. **D** Feature plots showing the spatial expression patterns and distribution of key marker genes within representative cell types. **E** Temporal expression patterns of selected marker genes during root callus development, analyzed using *LBD13P::GUS* and *GH3p::GUS* reporter lines, offering insights into their dynamic regulatory roles throughout the developmental process. (Scale bar: 1 mm)

In the case of root-derived callus, UMAP analysis identified nine cell clusters (Fig. 3A). We also identified the DEGs in each of clusters in root-derived callus samples (Table S3) and performed the GO and KEGG analysis for these DEGs (Fig. S2B and Table S4). The results indicate that intracellular transport is enriched in clusters 6 and 7 (Fig. S2B). Ribosome and ribonucleoprotein complexes are enriched in clusters 1 and 6 (Fig. S2B). The enrichment of membrane protein complexes in clusters 1, 6, and 7 (Fig. S2B). The MAPK signaling pathway and response to wounding are enriched in clusters 4 and 5 (Fig. S2B). The Sulfur compound metabolic process is enriched in clusters 8 and 9 (Fig. S2B).

The proportions of these clusters in each sample revealed that clusters 1, 2, and 3 were significantly enriched in root callus samples compared to root initial callus (Fig. 3B). Conversely, clusters 4, 5, and 8 were more prominent in the root initial callus and root callus greening stages (Fig. 3B). The dot_plot illustrates the expression levels of the selected representative marker genes within each cell cluster (Fig. 3C). Furthermore, the Feature_plot analysis of *GH3.3* and *LBD13* reveals their distribution and expression patterns across distinct cell populations (Fig. 3D). To investigate the expression patterns of *GH3.3* and *LBD13*, we generated Arabidopsis transgenic plants harboring GUS reporter genes driven by the respective endogenous promoters of these two genes (Fig. S3). Further analysis of the expression patterns of *GH3.3* (cluster 1) and *LBD13* (cluster 8) using GUS reporter lines showed that *GH3.3p::GUS* was predominantly expressed in late-stage (35-day) root-derived callus, whereas *LBD13p::GUS* was highly expressed before day 26 and markedly reduced in the mature callus (31-day) (Fig. 3E). These results suggest that the expression patterns of genes specific to each identified cell type align with their roles in callus development. Therefore, this method of annotating cell types based on selective expression of marker genes proves effective. However, due to the absence of a standard nomenclature for specific callus cell types, our annotations represent only the high-level expression of identified marker genes within these cell types. In other words, high-level expression of a specific marker gene can aid in determining the identity or developmental stage of callus cells.

### Integrated analysis of callus cells at different developmental stages in Arabidopsis leaves and roots

**Fig. 4:**
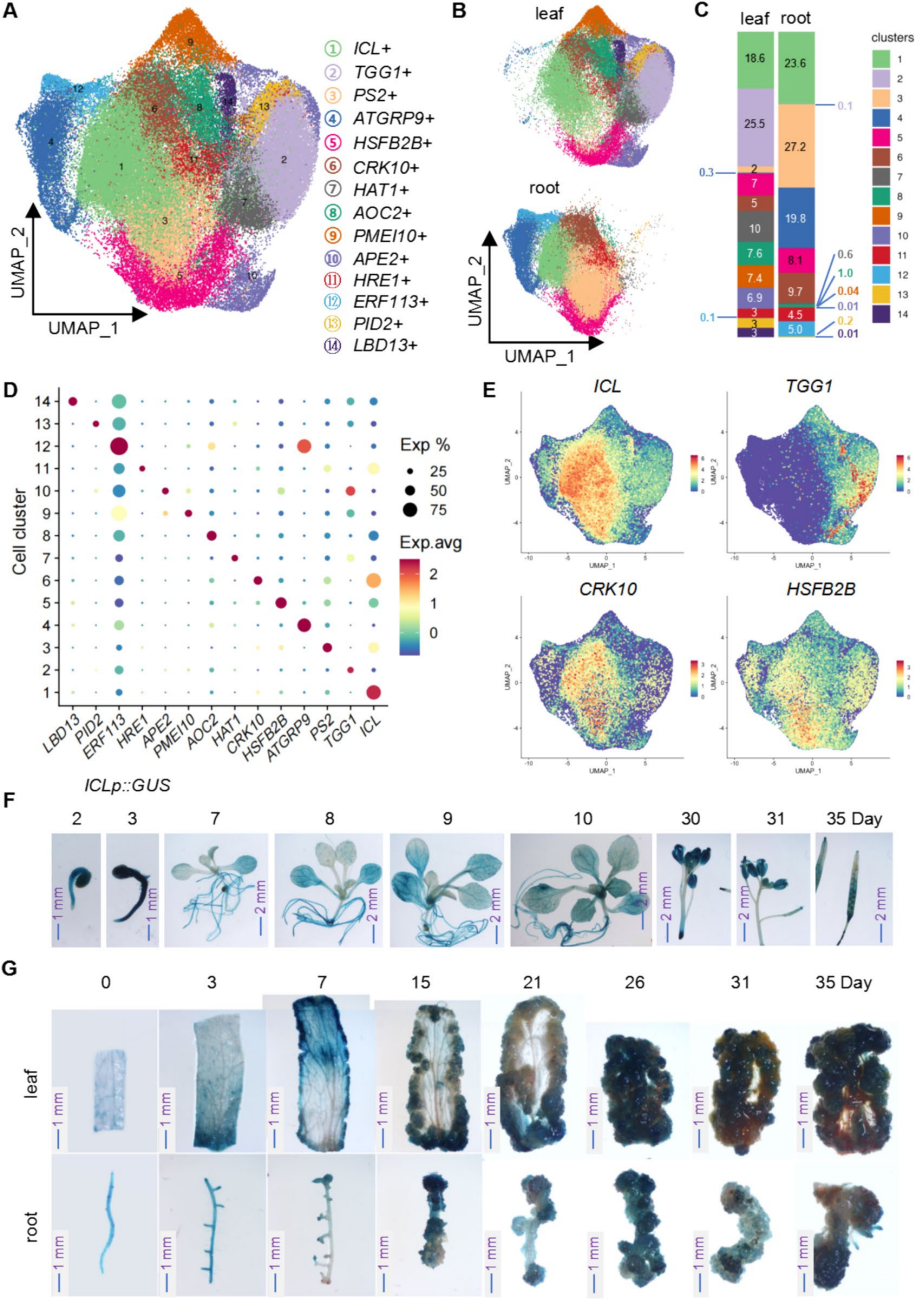
Integrated scRNA-seq analysis and cell type characterization of leaf- and root-derived callus. **A** UMAP projection of integrated single-cell transcriptomic data from both leaf- and root-derived callus, revealing 11 distinct cell clusters. Each cluster is defined by the specific expression of marker genes corresponding to unique cell types. **B** UMAP projection illustrating the spatial distribution of leaf- and root-derived callus cells within the integrated dataset. Left Panel: The distribution of cells from leaf-derived callus and root-derived callus in the UMAP space is shown. Right Panel: The stacked bar chart illustrates the proportional representation of cells from different clusters in leaf-derived callus and root-derived callus, with the numbers corresponding to the percentage of total cells within each sample. **C** Quantitative analysis of the relative proportions of different cell types in leaf- and root-derived callus tissues. **D** Dot plot showing the expression levels of key marker genes across the identified cell clusters and their associated cell types. **E** Feature plots visualizing the expression patterns and spatial distribution of key marker genes within representative cell types. **F** Expression patterns of *ICLp::GUS* at different developmental stages and in various tissues of Arabidopsis. (Scale bar: 1 mm or 2 mm). **G** Comparative analysis of *ICLp::GUS* and *GH3p::GUS* expression in leaf- and root-derived callus at different developmental stages. (Scale bar: 1 mm)

Given the morphological similarities between leaf- and root-derived callus during later stages of development, we conducted an integrated analysis of callus samples from both tissues (Fig. 4). UMAP projection revealed 11 distinct cell clusters (Fig. 4A). The DEGs in each of clusters in integrated root and leaf samples were identified (Table S5). Clusters 1, 5, and 6 were highly represented in both leaf and root samples, suggesting that these clusters correspond to mature callus cells (Fig. 4B and C). Subsequently, we performed dot_plot analysis on representative genes selected from each cell cluster. The results revealed that these representative genes exhibit highly specific expression patterns within their corresponding cell clusters (Fig. 4D). To further elucidate the specificity of gene expression within particular cell clusters, we analyzed the Feature_plots of four genes: *ICL*, *TTG1*, *CRK10*, and *HSFB2B* (Fig. 4E). The results indicated that these genes are expressed at higher levels within their respective cell clusters, underscoring the distinctiveness of their expression profiles. To investigate *ICL* expression in both seedlings and callus, we generated transgenic Arabidopsis lines expressing GUS reporters driven by the native *ICL* promoter (Fig. S3A). GUS staining analysis showed that *ICL* was expressed at varying levels across most developmental stages, with notably high expression in germinating seeds and inflorescences (Fig. 4F). In both leaf- and root-derived callus, *ICL* expression was elevated in the induced callus regions (7 days) and in mature callus (26–35 days), following similar expression trends in root callus (Fig. 4G). These findings suggest that ICL plays a prominent role throughout callus induction and development in both leaf and root tissues.

In addition to the shared cell types found in leaf and root callus, tissue-specific cell types were also identified. For instance, clusters 2, 7, 8, 9, 10, 13 and 14 were predominantly observed in leaf callus, while clusters 3, 4, 6, and 12 were largely confined to root callus. These results underscore the presence of distinct cell type differences between leaf- and root-derived callus, consistent with our morphological observations (Fig. 1B). During the early stages of callus development (7–15 days), subtle morphological discrepancies can be observed between callus cells derived from leaves and those originating from roots. However, as callus proliferation progresses (by day 26), these morphological differences become increasingly attenuated, resulting in a striking resemblance between the two cell types. This morphological convergence may reflect a parallelism in gene expression profiles, whereas cell types that retain pronounced morphological distinctions are likely to exhibit substantial transcriptomic divergence. Consequently, these variations in gene expression contribute to the emergence of distinct cell clusters in UMAP dimensionality reduction analysis.

### Pseudo-time analysis of callus formation and development

**Fig. 5:**
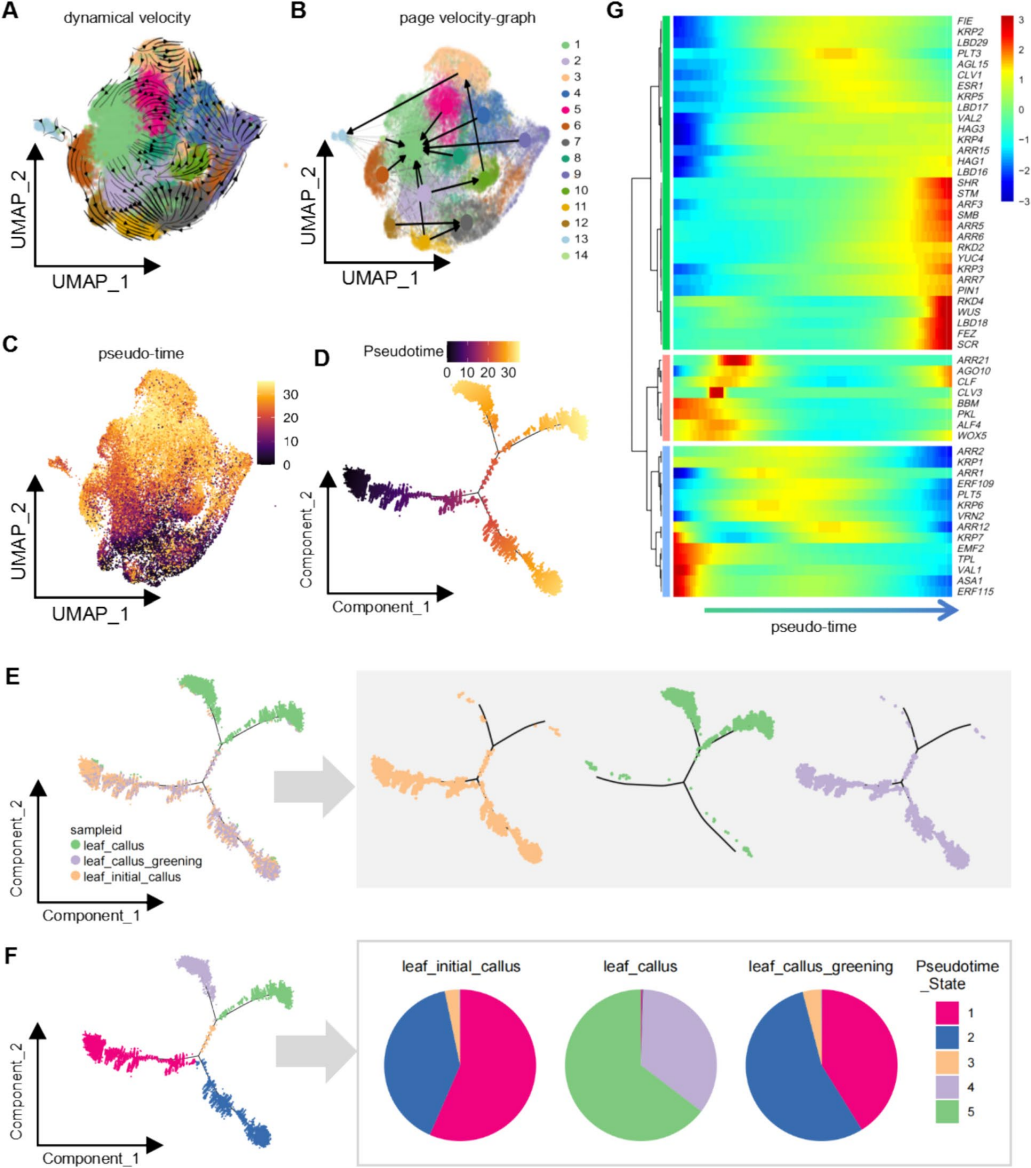
Dynamic analysis of leaf callus cell development. **A** RNA velocity analysis of leaf callus cells, where arrows indicate the inferred developmental trajectories from early to late stages. Different cell clusters are represented by distinct colors. **B** Partition-based graph abstraction (PAGA) velocity plot of leaf callus cells, showing the developmental trajectories from early to late stages. Arrows represent the direction of development, and colors denote various cell clusters. **C** Pseudo-time analysis of leaf callus cells displayed on a UMAP plot. Cells are color-coded from dark to light, corresponding to their progression through developmental stages from early to late. **D** Developmental trajectory plot of leaf callus cells, with colors representing distinct developmental stages across callus samples. **E** The cell_trajectory_color_by_sample. Left Panel: the distribution of cells from the three different developmental stages along the cell trajectory is depicted. Right Panel: the three plots on the right show the distribution of cells across the cell trajectory for each developmental stage sample. **F** The cell_trajectory_color_by_state. Left Panel: the distribution of cells from five distinct states along the cell trajectory is presented. Right Panel: the three pie charts on the right display the proportion of cells in each of the five states across the three developmental stage samples. **G** Heatmap showing dynamic expression patterns of key genes involved in callus development along the pseudo-time axis. Arrows indicate the progression of development from early to later stages

Distinct cell types within callus tissues exhibit notable developmental differences, particularly in terms of morphological reorganization (Fig. 1). To better understand the dynamic developmental trajectories of various cell types in leaf- and root-derived callus, we performed pseudo-time analysis using scRNA-seq data from both tissues. Scanning electron microscopy (SEM) revealed the rapid emergence of distinct cell morphologies during leaf callus induction, suggesting swift growth and cellular reorganization. To further investigate the developmental pathways of different callus cell types, we conducted RNA velocity analysis (Fig. 5A). The arrows in the velocity plot indicate the inferred developmental directions from early to late stages. Projecting these RNA velocity results onto a Partition-based graph abstraction (PAGA) velocity plot allowed the identification of several key developmental trajectories (Fig. 5B). Additionally, pseudo-time analysis across all cell populations (Fig. 5C) confirmed that these inferred trajectories were consistent with the RNA velocity trends.

Pseudo-temporal ordering of cells from different samples revealed a progression from initial leaf callus to mature leaf callus (Fig. 5D and E). Notably, cells from the initial leaf callus and leaf callus greening stages overlapped significantly in pseudo-time, likely due to their similar gene expression profiles (Fig. 5E). Consistent with the distribution pattern of cells along the pseudotime trajectory, the Pseudotime_state analysis revealed that state 1 and state 2, representing early developmental stages, predominated in the leaf_initial_callus, whereas state 4 and state 5 were more prevalent in the leaf_callus. Additionally, in the leaf_callus_greening, cells were primarily distributed in state 1 and state 2; however, a greater proportion of cells resided in state 2, in contrast to leaf_initial_callus, where state 1 was more dominant (Fig. 5F). As both cell types are undergoing rapid development and differentiation, their transcriptional profiles may more closely resemble one another than those of mature callus cells. Further analysis of genes regulating callus development revealed that genes promoting early developmental fates (e.g., auxin- and cytokinin-related genes) were lowly expressed in the early pseudo-time stages but increased rapidly as development progressed. Conversely, genes associated with callus formation and differentiation (e.g., *PLT3* and *ERF109*) exhibited increasing expression throughout pseudo-time, peaking in mid-developmental stages (Fig. 5 G). Genes involved in bud formation [e.g., BABY BOOM (*BBM*)] were predominantly expressed during the early stages of pseudo-time (Fig. 5 G). Overall, the expression patterns of these genes along the pseudo-time trajectory align with their established roles in developmental regulation.

**Fig. 6:**
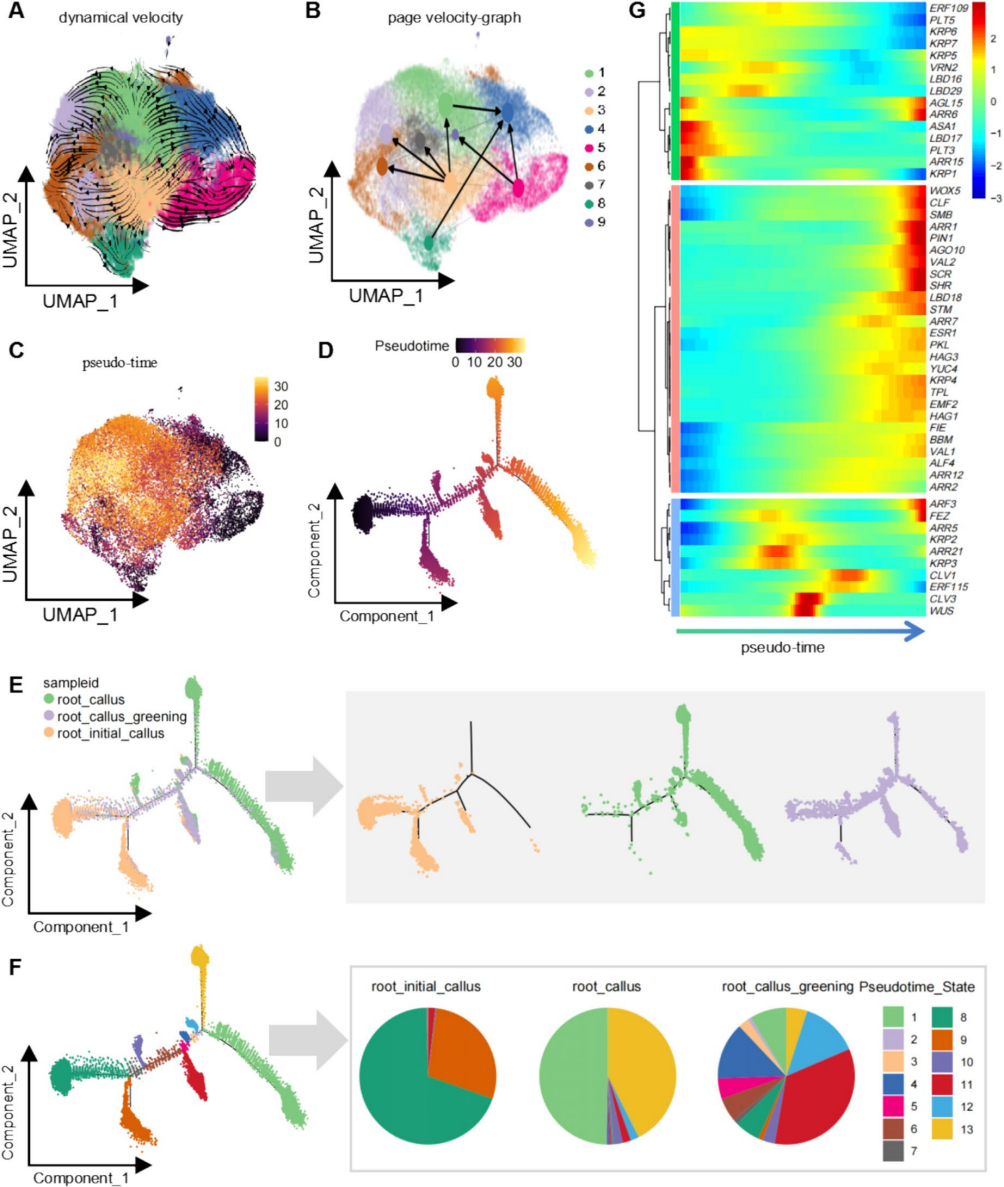
Dynamic analysis of root callus cell development. **A** Dynamic velocity analysis of root callus cells, with arrows indicating the trajectory of cellular development from early to late stages. Different colors denote distinct cell clusters. **B** PAGA velocity graph showing the results of the dynamic velocity analysis for root callus cells. Arrows indicate developmental trajectories from early to late stages, and different colors correspond to various cell clusters. **C** Pseudo-time analysis of root callus cells, visualized in a UMAP plot. Cells are color-coded from dark to light, reflecting their progression through developmental stages. **D** Developmental trajectory plot of root callus cells, where different colors represent cells at various developmental stages within the callus samples. **E** The cell_trajectory_color_by_sample. Left Panel: the distribution of cells from the three different developmental stages along the cell trajectory is depicted. Right Panel: the three plots on the right show the distribution of cells across the cell trajectory for each developmental stage sample. **F** The cell_trajectory_color_by_state. Left Panel: the distribution of cells from five distinct states along the cell trajectory is presented. Right Panel: the three pie charts on the right display the proportion of cells in each of the five states across the three developmental stage samples. **G** Heatmap depicting the dynamic expression patterns of key genes regulating callus development along the pseudo-time trajectory. Arrows indicate the direction of developmental progression from early to late stages

We subsequently performed RNA velocity analysis on root-derived callus (Fig. 6A). Consistent with the observations in leaf-derived callus, the PAGA velocity plot identified key developmental trajectories (Fig. 6B). Pseudo-time analysis corroborated these findings, showing congruent developmental patterns in UMAP projections, thereby confirming the robustness of the analysis (Fig. 6C). Pseudotemporal ordering revealed a trajectory from root_initial_callus to mature root_callus, with root_callus_greening cells situated between these stages (Fig. 6D). As seen in leaf_callus_greening, root_callus_greening cells exhibited intermediate developmental patterns, likely reflecting their rapid differentiation and growth (Fig. 6D).

We further examined the expression profiles of genes regulating callus development along the pseudo-time trajectory (Fig. 6A). As in leaf-derived callus, cytokinin-responsive genes were highly expressed during early stages but rapidly declined thereafter. Interestingly, several auxin-responsive genes displayed low expression at the early stages, followed by a sharp upregulation in the later stages of development (Fig. 6E). Similarly, genes involved in callus formation and development (e.g., *LBD* and *ERF109*) showed a gradual increase in expression along the pseudo-time trajectory, peaking in early to mid-developmental stages before decreasing rapidly (Fig. 6E).

Based on the results of pseudotemporal analysis, we identified cell clusters that emerge during the late stages of callus development and examined the biological functions associated with genes specifically expressed within these clusters. In the later phases of leaf-derived callus development, the predominant cell clusters were cluster 4 and cluster 5 (Fig. S4A). GO analysis revealed that genes uniquely expressed in these clusters were significantly enriched in biological processes related to environmental stress responses, including water stress, temperature fluctuations, oxidative stress, and wound response (Figs. S4B and C). Notably, genes in Cluster 5 were also enriched in pathways associated with hypoxia and osmotic stress, suggesting that these factors may play a role in regulating late-stage callus development (Fig. S4C). Furthermore, KEGG pathway analysis indicated that genes specifically expressed in clusters 4 and 5 were predominantly involved in metabolic regulatory pathways, such as oxidative phosphorylation, the tricarboxylic acid (TCA) cycle, and pyruvate metabolism, as well as processes related to environmental adaptation and immune defense (Figs. S4B and C). In root-derived callus, the dominant cluster at the late developmental stage was cluster 7 (Fig. S5A). GO analysis demonstrated that genes specifically expressed in cluster 7 were highly enriched in pathways associated with the ribosome, proteasome, and spliceosome, and were closely linked to ribonucleoprotein biogenesis and mRNA surveillance pathways (Fig. S5B). KEGG analysis further revealed that these genes were primarily involved in carbon metabolism, the TCA cycle, pyruvate metabolism, and cellular respiration (Fig. S5B). Additional analyses indicated that genes in cluster 7 also contributed to environmental stress responses, including hypoxia, salt stress, temperature fluctuations, and defense mechanisms (Fig. S5B).

### Transcriptional regulatory network analysis in Arabidopsis leaf and root callus cells

**Fig. 7:**
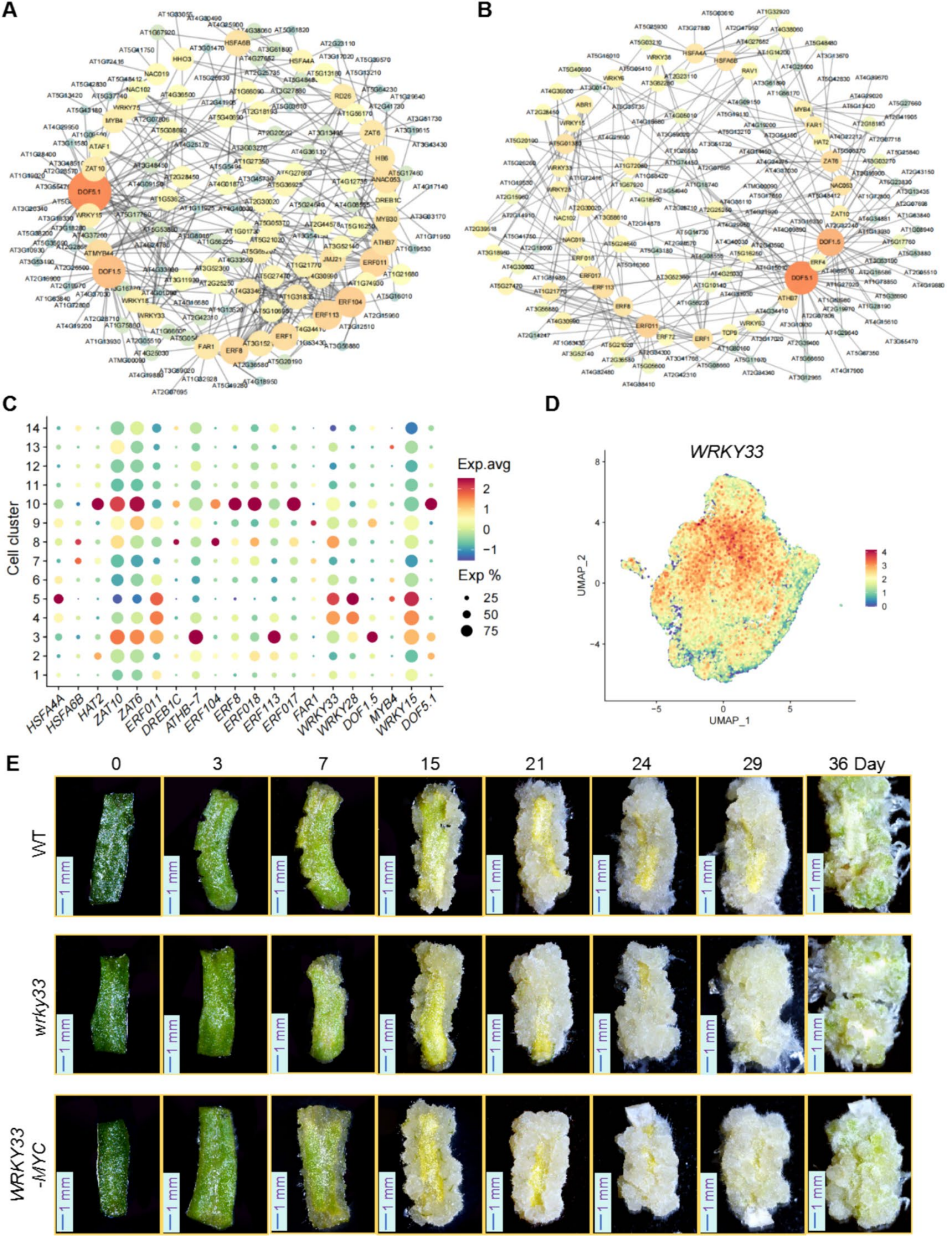
Transcriptional regulatory network analysis of differentially expressed genes in leaf callus cells at various developmental stages. **A** Transcriptional regulatory network analysis of differentially expressed genes between leaf_callus_greening and leaf_callus samples. **B** Transcriptional regulatory network analysis of differentially expressed genes between leaf_callus and leaf_initial_callus samples. **C** A dot plot illustrating the expression levels of the identified core transcription factors across different cell clusters. **D** Feature plot analysis depicting the expression pattern of *WRKY33*. **E** Phenotypic observations of leaf callus at various developmental stages in wild-type (WT), *wrky33*, and *35S::WRKY33-MYC *Arabidopsis lines. (Scale bar: 1 mm)

Transcription factors play a pivotal role in regulating cellular development. To identify transcription factors involved in leaf callus development, we first screened for DEGs between the leaf_callus_greening and leaf_callus samples (leaf_callus_greening vs. leaf_callus), as well as between leaf_callus and leaf_initial_callus samples (leaf_callus vs. leaf_initial_callus). Among the identified DEGs, we focused on transcription factors and their corresponding target genes involved in regulatory processes. Subsequently, we performed a transcriptional regulatory network analysis of these genes (Fig. 7A and B). The results indicated that transcription factors Such as DNA BINDING WITH ONE FINGER 5.1 (DOF5.1), DOF1.5, ETHYLENE RESPONSIVE FACTOR113 (ERF113), and WRKY33 regulate a relatively large number of target genes, underscoring their critical roles in orchestrating developmental processes at specific stages of leaf callus formation. Furthermore, we conducted dot plot analysis to assess the expression levels of these core transcription factors across various cell clusters (Fig. 7C). Figure 7D presents the feature plot of *WRKY33*, one of the representative transcription factors. Notably, the involvement of DOF5.1, DOF1.5, and ERF113 (Miyashima et al. 2019; Park et al. 2003, 2025) in regulating cell fate and callus development has been previously documented, while the potential role of WRKY33 in callus regulation remains to be characterized.

Expression analysis of *WRKY33* revealed its predominant expression in cluster 5 (Fig. 7D). Further analysis of the callus induction state in *wrky33* mutants and *35S::WRKY33-MYC* transgenic plants revealed that *wrky33* mutants exhibited significantly faster callus induction compared to WT plants, whereas *35S::WRKY33-MYC* plants displayed a callus development rate similar to that of WT but with slower greening (Fig. 7E and Fig. S6). These results imply that WRKY33 may negatively regulate callus development.

**Fig. 8:**
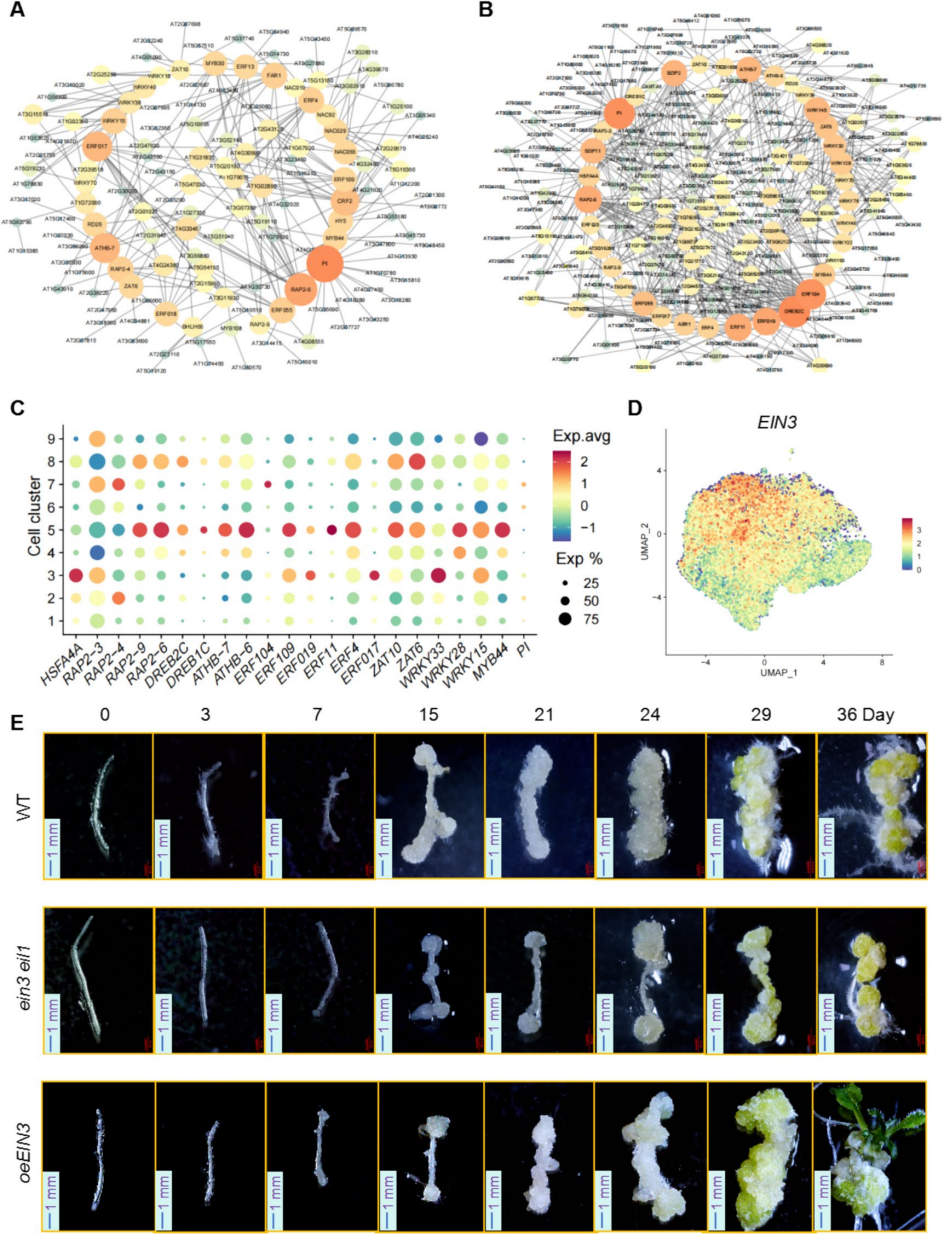
Transcriptional regulatory network analysis of differentially expressed genes in root callus cells at various developmental stages. **A** Transcriptional regulatory network analysis of differentially expressed genes between root_callus_greening and root_callus samples. **B** Transcriptional regulatory network analysis of differentially expressed genes between root_callus and root_initial_callus samples. **C** A dot plot displaying the expression levels of the identified core transcription factors across different cell clusters. **D** Feature plot analysis illustrating the expression pattern of *EIN3*. **E** Phenotypic observations of root callus at various developmental stages in wild-type (WT), *ein3 eil1*, and *oeEIN3 *Arabidopsis lines. (Scale bar: 1 mm)

Next, we conducted a differential expression analysis to compare genes in the root_callus_greening samples with those in the root-derived callus samples (root_callus_greening vs. root_callus) and between root_callus and root_initial_callus (root_callus vs. root_initial_callus). Among the identified DEGs, we further examined transcription factors and their respective target genes. Subsequently, a transcriptional regulatory network analysis was performed to investigate transcription factors and target genes specifically expressed during distinct developmental stages of root callus (Fig. 8). This analysis revealed that transcription factors such as PISTILLATA (PI), RELATED TO AP2 2–6 (RAP2-6), DEHYDRATION-RESPONSIVE ELEMENT BINDING PROTEIN 2 (DREB2C), and ERF104 (Fig. 8A and B), which are associated with the ethylene signaling pathway, play pivotal roles in regulating root callus development. Research has demonstrated that ETHYLENE INSENSITIVE 3 (EIN3) and ETHYLENE INSENSITIVE 3-like 1 (EIL1) play crucial roles in regulating the ethylene signaling pathway (Alonso et al. 2003). Feature plot analysis indicated that *EIN3* is predominantly expressed in cluster 1 (Fig. 8D), which corresponds to the late stage of root callus development. Despite its specific expression in cluster 1, given its central role in ethylene signaling regulation, we performed callus induction assays using the *ein3 eil1* double mutant and *EIN3*-overexpressing Arabidopsis lines (*oeEIN3*) (Fig. 8E). This allowed us to further investigate the function of ethylene signaling in root callus induction and development. The results indicated that the induction rate of root callus in the *ein3 eil1* double mutant was significantly slower than that in the WT, whereas the *oeEIN3* exhibited a markedly faster callus induction rate compared to WT, particularly in terms of greening and bud emergence (Fig. 8E). By day 36 of the induction culture, the formation of new buds was clearly visible (Fig. 8E).

### Identification of core regulatory factors in callus cells at various developmental stages in Arabidopsis leaves and roots

**Fig. 9:**
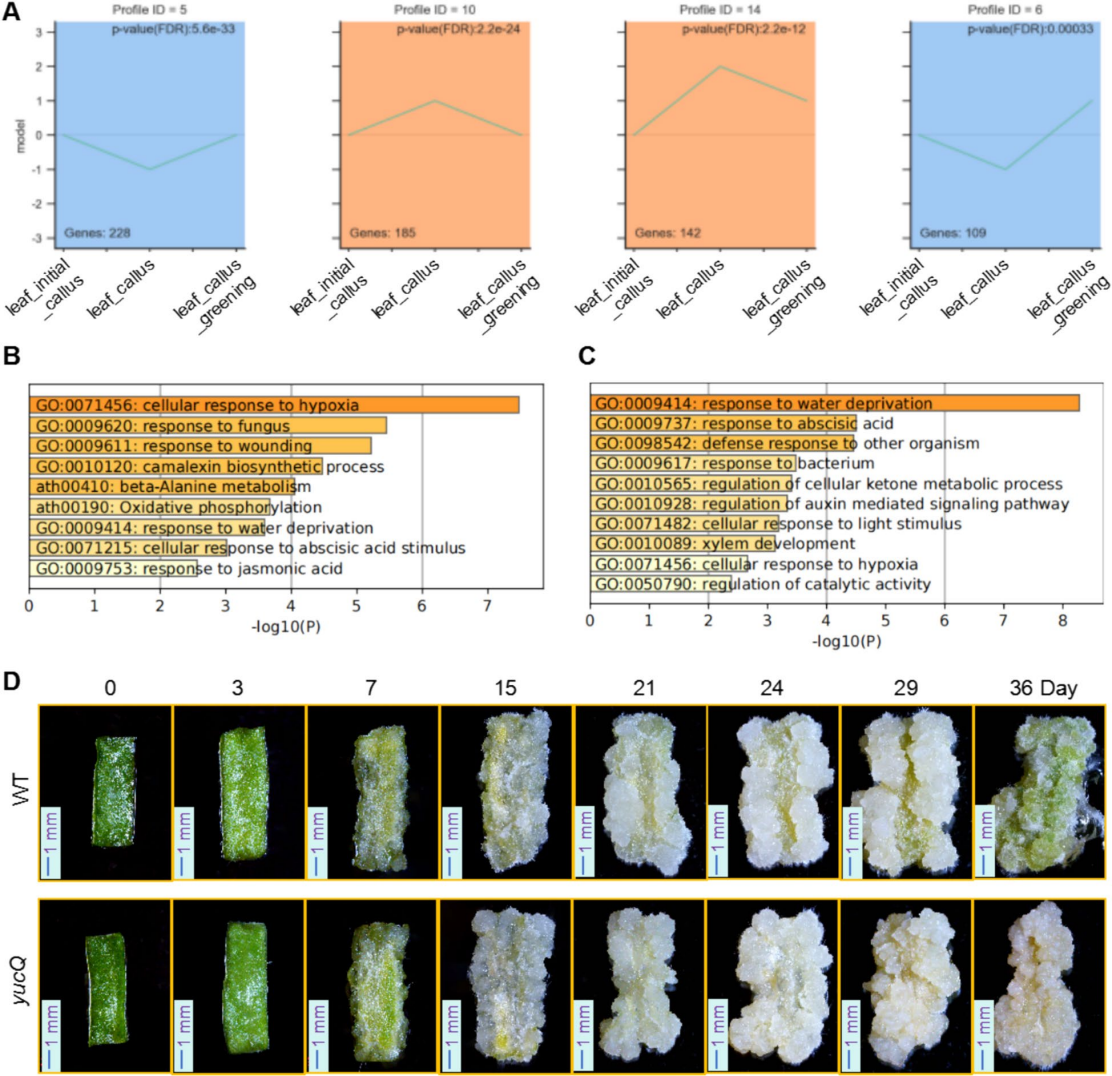
Cluster series analysis of gene expression in leaf callus cells at distinct developmental stages. **A** The expression trends of stage-specific genes during the transition from leaf_initial_callus to leaf_callus and leaf_callus_greening. **B** Gene Ontology (GO) analysis of genes with significantly elevated expression in leaf_callus. **C** GO analysis of genes with significantly elevated expression in leaf_callus_greening. **D** Phenotypic analysis of growth patterns in leaf callus at different developmental stages in both WT and the *yucQ* quadruple mutant lines. (Scale bar: 1 mm)

To identify genes with pivotal regulatory roles at specific developmental stages, we first conducted a differential expression analysis across various stages by identified the DEGs between leaf_callus-vs-leaf_initial_callus (Table S6) and the DEGs between leaf_callus_greening-vs-leaf_callus (Table S7). A subsequent trend analysis with Short Time-Series Expression Miner (STEM) on these differentially expressed genes identified those with significantly elevated expression during the leaf callus and leaf callus greening stages (Fig. 9A and Table S8). Gene Ontology (GO) and Kyoto Encyclopedia of Genes and Genomes (KEGG) analyses were employed to investigate the biological processes associated with these genes (Fig. 9B and C). These analyses revealed that several genes specifically expressed during the greening phase of leaf callus are involved in the regulation of auxin signaling pathways.

Cheng et al. (2007) demonstrated that the flavin monooxygenases of the YUC family play a pivotal role in auxin biosynthesis, serving as a crucial source of this phytohormone during both embryogenesis and post-embryonic organ formation in *Arabidopsis* *thaliana* (Chen et al. 2016). Moreover, YUC-mediated auxin synthesis is intimately linked to its polar transport (Cheng et al. 2007). Specifically, *YUC1* and *YUC4* are expressed throughout embryonic development, exhibiting overlapping expression patterns with *YUC10* and *YUC11*(Cheng et al. 2007). Collectively, these genes contribute to the establishment of the embryonic basal region and the formation of both embryonic and post-embryonic organs (Chen et al. 2016; Cheng et al. 2007). Notably, the quadruple mutant *yuc1 yuc4 yuc10 yuc11* (*yucQ*) exhibits a severe developmental defect, failing to form both the hypocotyl and root meristem— a phenotype reminiscent of mutations in MP and TIR1/AFB class genes, which are also critical for embryogenesis (Chen et al. 2016). Furthermore, the *YUC* gene family interacts with key regulators of polar auxin transport, such as PIN1 and AUX1, underscoring their indispensable role in leaf and floral development (Cheng et al. 2007). To further validate the role of auxin signaling in callus tissue greening, we performed callus induction experiments using *yucQ* mutants and WT plants. Compared to WT, the *yucQ* mutants showed no significant difference in callus induction and growth rates (Fig. 9D). However, after 24 days in greening culture, the *yucQ* mutants displayed markedly reduced callus greening (Fig. 9D). These findings suggest that YUC-mediated auxin biosynthesis is essential for leaf callus greening.

**Fig. 10:**
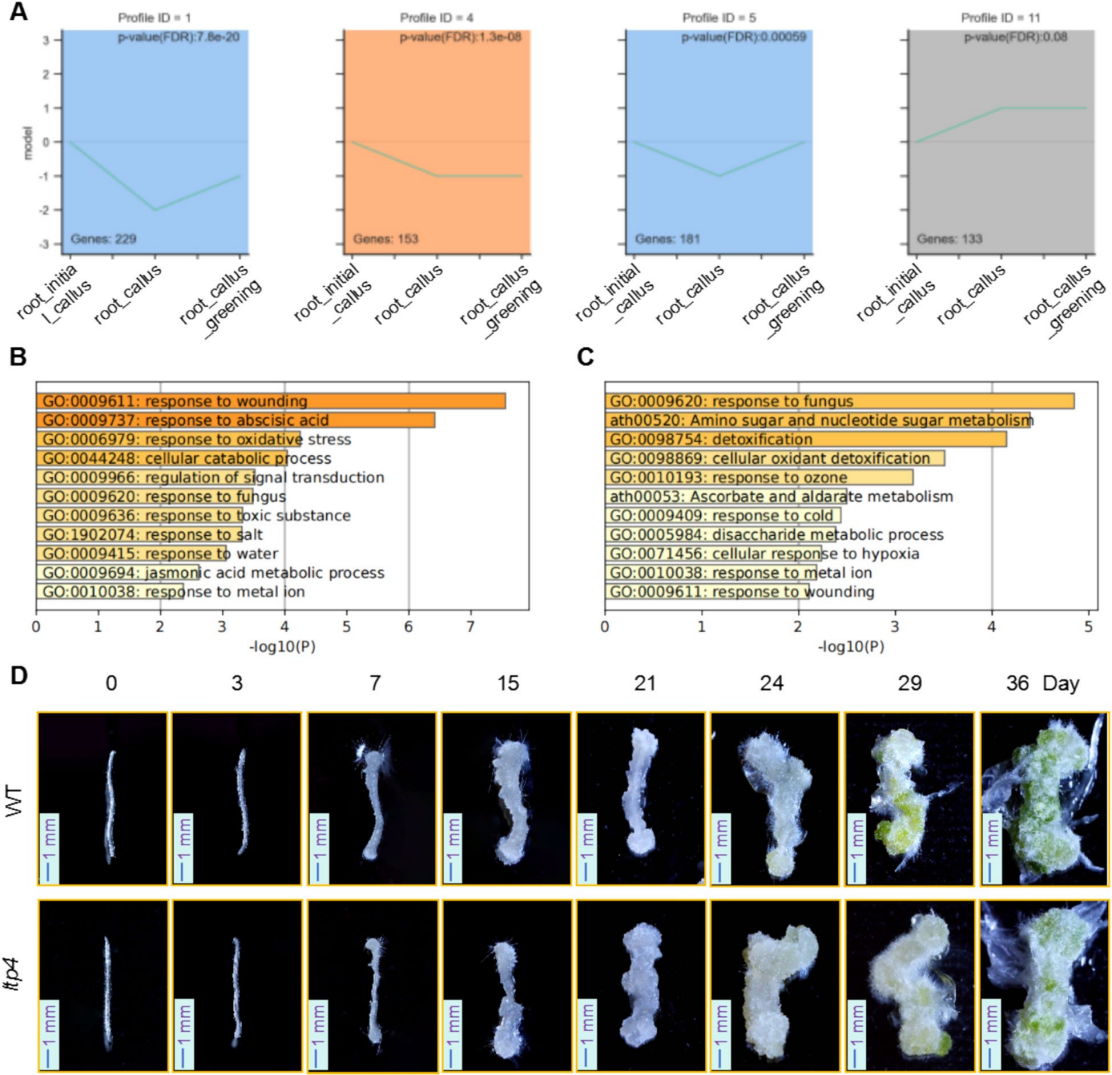
Cluster series analysis of gene expression in root callus cells at distinct developmental stages. **A** The expression trends of stage-specific genes during the transition from root_initial_callus to root_callus and root_callus_greening. **B** GO analysis of genes with significantly elevated expression in root_callus. **C** GO analysis of genes with significantly elevated expression in root_callus_greening. **D** Phenotypic analysis of growth patterns in root callus at different developmental stages in both WT and the *ltp4* mutant lines. (Scale bar: 1 mm)

Similarly, we identified the DEGs between root_callus-vs-root_initial_callus (Table S9) and the DEGs between root_callus_greening-vs-root_callus (Table S10). Then we conducted a STEM analysis with these DEGs (Fig. 10A and Table S11). GO and KEGG analyses were used to explore the biological processes associated with genes specifically expressed during root callus and root callus greening stages (Fig. 10B and C). The results revealed that genes specifically expressed during root callus development were associated with oxidative stress responses, while those more highly expressed during the root callus greening stage were related to abscisic acid (ABA) signaling.

*LTP3* plays a pivotal role in plant immunity by modulating the abscisic acid (ABA) pathway and exhibits functional redundancy with its homolog *LTP4* (Gao et al. 2016). Studies have demonstrated that the overexpression of *LTP3* (*LTP3-OX*) markedly upregulates the expression of ABA biosynthetic genes (*NCED3* and *AAO3*), leading to elevated ABA levels, while concurrently downregulating salicylic acid (SA)-related genes (*ICS1* and *PR1*), thereby suppressing SA accumulation and increasing plant susceptibility to pathogens (Gao et al. 2016). However, the loss-of-function mutant *ltp3-1* does not significantly alter the ABA pathway. Further investigation revealed that the *ltp3/ltp4* double mutant exhibits reduced pathogen susceptibility, accompanied by the downregulation of ABA biosynthetic genes, confirming that LTP3 and LTP4 collaboratively regulate ABA-SA homeostasis to modulate plant immune responses (Gao et al. 2016). To investigate the role of ABA signaling in root callus greening, we conducted callus induction experiments on *ltp4* (lipid transfer protein 4) mutants and WT plants. The greening rate of root callus (from day 24 to day 36) in the *ltp4* mutants was significantly slower than that in WT (Fig. 10D), suggesting that LTP4-mediated ABA signaling is crucial for root callus greening.

### Impact of environmental factors on callus development

Our analysis revealed a strong correlation between callus development and various environmental factors, consistent with previous studies emphasizing the role of dedifferentiation and redifferentiation in plant regeneration under injury or environmental stress conditions (Sugimoto et al. 2019). Through STEM analysis, we identified genes that are specifically highly expressed in leaf_callus and leaf_callus_greening. Subsequent GO and KEGG pathway analyses revealed that genes uniquely expressed in both leaf_callus and leaf_callus_greening are significantly enriched in pathways related to cellular response to hypoxia. Moreover, genes specifically expressed in leaf_callus_greening are markedly enriched in pathways associated with cellular response to light stimulus (Fig. 9B and C). To further explore the influence of environmental conditions on callus development, we examined the effects of hypoxia, light, and salinity on this process.

Initial results indicated a significant enrichment of hypoxia-responsive genes in leaf callus tissue. To assess the impact of hypoxia on callus development, we subjected WT callus tissue to hypoxic conditions, using callus grown under normal conditions as a control. As illustrated in Figs. S7A and B, callus tissue exposed to hypoxia exhibited earlier and more pronounced chlorosis than control tissue. After seven days of treatment, noticeable differences in color were observed between the two groups (Figs. S7A and B). By day 15, white cell clusters had formed in the control group, but there was no significant difference in sample weight at this stage (Figs. S7A and B). By day 21, the weights of callus from both groups remained statistically similar, although callus grown under hypoxic conditions showed a slightly accelerated growth rate (Figs. S5C and D). These findings suggest that hypoxia facilitates callus induction and development.

PROTEOLYSIS 6 (PRT6) is a critical E3 ligase that regulates the N-end rule pathways and plays a pivotal role in modulating plant responses to hypoxia and other environmental stresses (Garzon et al. 2007). As a component of the ubiquitin–proteasome system, PRT6 mediates the degradation of proteins with an amino-terminal arginine (Arg) residue, thereby controlling the stability of key substrates such as ETHYLENE RESPONSE FACTOR VIIs (ERFVII) and LITTLE ZIPPER 2(Holdsworth et al. 2020). These substrates are involved in plant responses to stressors such as flooding, salinity, and vernalization. Under normal conditions, PRT6 targets these transcription factors for degradation, but under stress conditions like reduced oxygen or nitric oxide levels, the activity of PRT6 is reduced, stabilizing these proteins and allowing them to regulate critical developmental processes and stress responses (Holdsworth et al. 2020). In addition to its role in stress adaptation, PRT6 also influences hypoxia responses by targeting proteins such as HYPOXIA-RESPONSIVE ERF2 (HRE2) for degradation (Zhang et al. 2024). The *prt6* mutant exhibit enhanced stability of hypoxia-related proteins, leading to an upregulation of hypoxia-responsive genes and altered root transcriptomes (Zhang et al. 2024). To investigate whether hypoxia signaling affects the development of callus tissue, we investigated callus development in the *prt6* mutant under normal growth conditions. As shown in Fig. S8A and B, callus development in *prt6* was slightly slower than in WT at all stages. During the initiation and proliferation phases, *prt6* exhibited lower callus weight compared to WT (Fig. S8C and D). Additionally, the root-derived callus of *prt6* displayed a slower rate of greening during the greening phase compared with that of WT (Figs. S8A and B). These results suggest that PRT6-mediated hypoxia signaling plays a crucial role in regulating callus development.

### Light as a key regulator of callus formation

Our previous analyses highlight the critical role of light in callus development (Fig. 9C). Light signaling has been shown to regulate a wide range of plant growth and developmental processes, with CONSTITUTIVE PHOTOMORPHOGENIC 1 (COP1) identified as a central regulator of photomorphogenesis. To further investigate the role of light in callus formation, we examined callus development in *cop1* mutants.

As depicted in Fig. S9A, after three days of culture, both *cop1* and WT leaf samples began to show signs of yellowing. By day seven, *cop1* leaves exhibited slightly less chlorosis compared to WT (Figs. S9A and B), while root callus development remained comparable between the two genotypes. By day fifteen, WT leaves and roots had entered the proliferation phase, producing typical white callus tissue (Fig. S9A). In contrast, *cop1* leaves did not generate white callus but instead turned yellow–brown (Fig. S9B). Although *cop1* mutant leaves failed to form the typical white callus, regeneration buds were observed at the wound sites (Fig. S9B). In *cop1* root tissue, callus formation was restricted to the wound margins. Further analysis (Figs. S9C and D) revealed that both leaf and root callus tissues from *cop1* mutants were significantly lighter in weight than those from WT. These results suggest that COP1 regulates both the initiation and proliferation of callus tissue.

We next performed callus induction experiments using the ELONGATED HYPOCOTYL 5 (*hy5*) mutant, which encodes the downstream transcriptional regulator of COP1. During the early stages of callus formation, no phenotypic differences were observed between *hy5* and WT calli (Figs. S10A and B). However, in the proliferation stage, *hy5* leaf callus showed no significant differences compared to WT, but fewer white hair-like structures were observed in *hy5* root callus between days 15 and 21 (Figs. S10A and B). By day 21, *hy5* calli exhibited a smoother surface relative to WT (Figs. S10A and B). During the greening phase, *hy5* calli displayed delayed greening compared to WT (Figs. S10A and B). Further analysis revealed that *hy5* root calli had a diminished capacity to induce new shoots, although root induction was relatively robust (Figs. S10A and B). Weight measurements over the 21-day period (Figs. S10C and D) indicated no significant difference between *hy5* and WT calli throughout the experiment. These findings suggest that HY5 predominantly regulates the greening phase of callus development.

To investigate the molecular basis of HY5-mediated regulation of callus development and greening, we performed quantitative reverse transcription polymerase chain reaction (qRT-PCR) to analyze the expression of *LIGHT-HARVESTING CHLOROPHYLL B-BINDING 2* (*LHCB2*), *PETAL LOSS* (*PTL*), and *WOX5* in *hy5* and WT callus tissues at days 24, 29, and 36 (Fig. S11). *LHCB*, a marker for chloroplast development, reflects the greening process, while *PTL* and *WOX5* are markers of embryonic cells, indicating bud regeneration. As shown in Figs. S11A–F, *LHCB2* expression was significantly reduced in both root and leaf callus of *hy5* compare with that of WT. In leaf callus, *PTL* and *WOX5* expression levels were lower in *hy5* than in WT at day 24, but significantly increased during the later stages of greening (29 and 36 day) (Figs. S11A, C and E), suggesting an accumulation of embryonic cells in mutant callus under prolonged light exposure. In contrast, in root callus, *WOX5* expression remained consistently lower than WT throughout the greening phase (36 day) (Figs. S9B, D and F), and *PTL* expression was markedly reduced in the late greening phase (36 day), indicating a diminished presence of embryonic cells in *hy5* root callus (Figs. S11F). These results highlight the central role of HY5 in mediating light-dependent signaling pathways during the greening phase of callus development.

### Moderate salt stress promotes the initiation of cellular regeneration

Through STEM analysis, we identified genes that are specifically highly expressed in root_callus and root_callus_greening. Subsequent GO and KEGG pathway analyses revealed that genes uniquely expressed in root_callus and root_callus_greening are significantly enriched in pathways related to response to wounding and response to fungus. In contrast, genes specifically expressed in leaf_callus are notably enriched in pathways associated with response to oxidative stress, response to salt, and response to abscisic acid (Fig. 10B and C). These results suggested that stress factors, such as oxidative oxidative stress or salt stress can influence callus tissue development. To further investigate the role of salt stress in this process, we exposed WT callus tissue to either normal conditions (control) or 50 mM NaCl treatment. As illustrated in Figs. S12A and B, phenotypic observations indicated that salt-treated leaves exhibited earlier and more pronounced yellowing compared to the control group, thereby triggering the initiation of regenerative responses. Additionally, after seven days, the leaf callus from the salt-treated group displayed a significant increase in weight compared to the control, while the root callus under salt stress demonstrated notable differences by day three (Figs. S12C and D). These findings suggest that salt stress plays a critical role in initiating callus formation. Regarding growth rate, callus tissue subjected to salt treatment exhibited a slight acceleration compared to the control group (Figs. S12A, C, D), further indicating that moderate salt stress enhances the initiation and early development of callus tissue.

Studies have demonstrated that RRTF1 plays important roles in regulating ROS production and ROS-responsive gene expression (Wang et al. 2020) and exhibits tissue-specific expression in roots under saline conditions, with its promoter not only driving initial transcriptional activation but also orchestrating a subsequent adaptive phase (Soliman and Meyer 2019). Notably, despite the persistence of salt stress, gene expression levels ultimately revert to baseline (Soliman and Meyer 2019). Further analysis indicates that prolonged high expression of RRTF1 exerts deleterious effects on plant viability (Soliman and Meyer 2019). Therefore, we also conducted callus culture experiments with the *REDOX RESPONSIVE TRANSCRIPTION FACTOR 1* (*rrtf1*) mutant, to check whether RRTF1 is involved in regulating the development of callus. The results (Fig. S13) revealed that during the early developmental stages, *rrtf1* callus showed no significant differences in growth rate compared to WT. However, during the greening phase, the *rrtf1* mutant produced significantly more new roots and exhibited a faster greening process than WT. This observation suggests that *rrtf1* callus is more sensitive to salt stress during the greening phase, supporting the findings presented in Fig. S13A at 36 days after treatment.

## Discussion

We utilized single-cell RNA sequencing (scRNA-seq) to construct a developmental lineage of callus tissue derived from Arabidopsis leaf and root explants. Our analysis highlighted the dynamic transcriptional changes occurring in both explants, characterized the cellular types at various developmental stages, identified key marker genes involved in callus formation and development, and explored how external environmental factors influence tissue regeneration.

### Construction of cellular developmental maps for leaf and root callus tissue

Callus tissue is typically described as an amorphous aggregation of cells lacking clear structural organization, formed through the processes of cellular dedifferentiation and redifferentiation. Due to morphological changes that occur across different developmental stages, callus consists of several distinct cell types, each with a unique gene expression profile (Figs. 1, 2, and 3). Therefore, identifying and analyzing these cell types is critical for unraveling the regulatory mechanisms that govern callus development. Using scRNA-seq, we performed an in-depth analysis of the callus developmental process (Fig. 1). UMAP analysis revealed distinct cell clusters in both leaf and root explants, characterized by unique gene expression patterns (Figs. 2 and 3). Additionally, we identified clusters exhibiting stage-specific gene expression profiles across three developmental phases (Figs. 2 and 3). Given the limited research on callus cell types and the absence of established marker genes for their annotation, we relied on genes or proteins with specific expression patterns to annotate the corresponding cell types (Figs. 2, 3, and 4).

Historically, it has been suggested that callus tissue primarily arises from root meristematic cells. However, our findings highlight significant differences between leaf- and root-derived callus in terms of marker genes, distribution patterns, and developmental trajectories (Figs. 2, 3, 4, 5, and 6), indicating that callus originating from different explants exhibits distinct gene expression profiles (Fig. 4). To further characterize the gene expression patterns at various developmental stages, we performed DEGs analysis and GO analysis to identify the key biological processes in which these genes are involved across different cell types (Fig. S2). Collectively, our study presents a comprehensive cellular atlas of callus development in *Arabidopsis* leaves and roots, revealing the gene expression dynamics throughout the three developmental stages of explants (Figs. 2, 3, and 4). This atlas serves as a valuable resource for future studies. However, some limitations remain, and further research is necessary to refine the identification, annotation, and functional analysis of specific cell clusters within callus tissue and to better understand their developmental regulatory mechanisms.

### Identification of marker genes affecting callus development

Following the annotation of cell types within callus tissue, we identified three marker genes—*ISOCITRATE LYASE* (*ICL*), *GH3.3*, and *LBD13*—to investigate their expressing patterns during callus development. GUS staining of transgenic plants showed that these genes were expressed in seedlings as early as two days post-germination (Fig. S3). *ICL* and *GH3.3* were initially concentrated in cotyledons and roots (Fig. 4), with expression later expanding throughout the plant. In contrast, the expression of *LBD13* gradually decreased over time (Fig. S3). Notably, *ICL* was also expressed in flowers and pods (Fig. S2). These findings suggest that *ICL*, *GH3.3*, and *LBD13* exhibit strong expression during early seedling development and play distinct roles in various tissues and organs. Given that early seedling cells possess significant pluripotency (Luo et al. 2016), it is plausible that these marker genes contribute to the dedifferentiation process.

We then performed callus culture experiments to further examine the involvement of these genes in callus initiation. Each gene exhibited distinct expression patterns at different developmental stages and in specific tissues within the callus (Figs. 2, 3, and 4), aligning with their dynamic expression profiles. Although all three genes are implicated in the regulation of callus initiation, *LBD13* displayed a notably different expression pattern in leaf callus compared to *ICL* and *GH3.3* (Figs. 2, 3, and 4). While *LBD13* was uniformly expressed across the entire leaf surface, *ICL* and *GH3.3* were predominantly localized at the leaf margins (Figs. 2, 3, and 4). This divergence in expression patterns suggests that these genes may be governed by different molecular regulatory mechanisms (Sugimoto et al. 2019). In conclusion, we have identified *ICL*, *GH3.3*, and *LBD13* as key marker genes involved in callus development, making them potential targets for further studies on plant regeneration.

### Influence of external factors on the development of leaf and root callus tissue

GO analysis of DEGs identified several external environmental factors that likely play critical roles in callus tissue development. Our experimental results suggest that, under standard growth conditions, many stem cells and genes expressed within their microenvironment respond to a variety of stressors and stress-related hormones (Zeng et al. 2021) (Fig. S2). Notably, genes involved in jasmonic acid, ethylene, and fatty acid metabolism were significantly enriched in our GO analysis (Fig. S2). Both jasmonic acid (Zhang et al. 2019) and ethylene (Jha et al. 2007; Lima et al. 2009; Wang et al. 2018) are well-established regulators of callus induction. For instance, jasmonic acid-mediated injury signals promote callus formation by modulating the expression of WIND genes, which, in turn, activate downstream cytokinin responses (Iwase et al. 2011). Additionally, long-chain fatty acids have been implicated in the regulation of callus formation (Shang et al. 2016; Trinh et al. 2019).

The STEM analysis revealed that adverse environmental conditions, such as hypoxia and salinity, promote callus development (Figs. 9 and 10). Specifically, we observed that low-oxygen treatment stimulates the development of callus (Fig. S7). Similarly, salt stress appears to disrupt normal cell division by facilitating the separation of intercellular contacts, thereby accelerating callus development (Fig. S12). In line with these findings, although the *rrtf* mutant did not significantly affect the rate of callus development, it exhibited a faster greening rate in later stages of development compared to the WT (Fig. S13). Furthermore, our study demonstrated that COP1-mediated light signaling inhibits callus proliferation (Fig. S9). While early and mid-stage plant tissue cultures are typically maintained in dark to promote rapid callus growth. However, based on the effects of *hy5* mutant on the greening of callus, we found that light signals is crucial during later stages to promote the greening of callus tissue (Fig. S10). Based on quantitative gene expression analysis on the chloroplast development-related gene *LHCB1.2*, as well as the callus development-related genes *PLT* and *WOX5*, we propose that once callus greening commences, its proliferation is suppressed, with energy redirected toward cellular differentiation and plant regeneration (Fig. S11).

## Conclusion

In this study, we elucidated the dynamic developmental profiles and transcriptomic characteristics of callus cells during plant regeneration in Arabidopsis. The application of scRNA-seq has allowed us to dissect the cellular heterogeneity within callus tissues at three critical developmental stages: initiation, proliferation, and greening. By integrating transgenic Arabidopsis lines and performing pseudotime analysis, we successfully traced the developmental trajectories of these cells, illuminating their differentiation pathways and the roles of pivotal regulatory genes in callus formation. Additionally, our GO analysis demonstrated significant enrichment of genes responsive to environmental factors, such as oxygen, light, and salinity, emphasizing the intricate interplay between external conditions and cellular responses during regeneration. This nuanced understanding of how environmental factors influence callus development not only enhances our knowledge of plant regenerative mechanisms but also provides a foundation for optimizing tissue culture protocols and improving the regenerative capacities of crop species. In conclusion, this study provides new insights into the role of external factors in plant tissue regeneration and offers practical strategies for optimizing plant tissue culture techniques. Specifically, applying targeted external stimuli at early and late stages of cultivation may trigger dedifferentiation or redifferentiation processes, while ensuring adequate nutrition and maintaining dark conditions during the intermediate phase to support rapid callus proliferation.

## Materials and methods

### Callus tissue cultivation from Arabidopsis leaf and root tissues

The WT Arabidopsis seeds were placed in 1.5 mL Eppendorf (EP) tubes and Surface-sterilized under a clean bench. The seeds were sequentially treated with 1 mL of 75% ethanol for 3 min, followed by 0.5 mL of 10% sodium hypochlorite solution for 10 min. Following sterilization, the seeds were rinsed 5–8 times with sterile water to remove any remaining sodium hypochlorite. The sterilized seeds were Subsequently sown on 1/2 MS solid medium plates and stratified at 4 °C for 2–3 days. The plates were then transferred to a 23 °C climate chamber in a vertical orientation, where seedlings were cultured for one week.

Under sterile conditions, the seedlings were transferred to 1/2 MS liquid medium and grown under long-day conditions (16-h light/8-h dark) for 2–3 weeks. Mature rosette leaves of similar size were selected and cut into strips approximately 5 mm in length and 2 mm in width using sterile scissors. The leaf segments were inoculated onto callus induction medium and incubated in darkness at 23 °C for approximately 21 days, with periodic monitoring and photographic documentation of callus formation. Root explants (5 mm) were prepared using the same protocol. For greening, leaf- or root-derived callus was transferred to greening medium and cultured under long-day conditions at 23 °C for 1–2 weeks, with periodic observation and imaging to document greening progress.

### Sample collection and protoplast isolation

Samples were collected from the initiation, formation, and greening stages of Arabidopsis WT-derived callus. Tissues were sectioned into small fragments and immersed in a freshly prepared enzyme solution, followed by 10 min of vacuum infiltration. Protoplast release was promoted by low-speed shaking in darkness for several hours. The isolated cells were washed twice with buffer to remove residual magnesium ions (Mg^2+^) and then filtered sequentially through 70 µm and 40 µm cell strainers for purification. Trypan blue staining assessed cell viability, and a hemocytometer was used to measure cell concentration. A 0.4% trypan blue solution was mixed with the protoplast Suspension at a 1:9 ratio, stained for 3–5 min, and applied to a hemocytometer for microscopic counting. Counting across 4–5 squares determined debris rate, cell viability, and concentration. Samples were used in Subsequent experiments if debris rate was below 5%, cell viability exceeded 85%, and concentration was above 800 cells/µL.

### scRNA-seq sequencing analysis

The freshly isolated protoplasts were adjusted to a concentration of 700–1200 cells/µL, following the 10 × Genomics Chromium Next GEM Single Cell 3’Kit v3.1 protocol. Initially, Master Mix bound individual cells with GEMs and barcodes. Subsequently, the Master Mix with cell Suspension, Gel Beads, and Partitioning Oil were loaded into a single-cell chip G, sealed with a 10× Gasket, and processed in the 10 × Genomics instrument for an 18-min run. Next, GEMs transfer and GEMs-RT incubation were conducted using PCR. After PCR, GEMs-RT products were purified and amplified into cDNA, which was then quality-controlled and quantified. cDNA meeting quality standards underwent fragmentation, end-repair, A-tailing, and selective enrichment to create high-quality libraries for high-throughput sequencing.

### Gene quantification, quality control, and data preprocessing

Raw data were analyzed using the Cell Ranger software by 10 × Genomics, which performs quality statistics and aligns data to the reference genome. This software identifies cellular barcodes and UMI tags for individual mRNA molecules in each cell, allowing for quantitative analysis that provides high-quality metrics such as cell counts, median genes, and sequencing saturation. Based on Cell Ranger’s preliminary quality metrics, subsequent data processing was conducted using the Seurat package. Most cells’gene expression levels, UMI counts, and mitochondrial gene expression cluster within expected ranges, allowing distribution modeling to identify and exclude outliers. Low-quality doublets, multiplets, and dead cells were excluded to refine the dataset further.

### Dimensionality reduction, clustering, and marker gene identification

Cells with similar gene expression profiles were grouped through clustering based on transcriptional data. The transcriptomic data were structured as an M × N-dimensional matrix, with genes in rows and cells in columns. Given the computational intensity and potential for suboptimal clustering results in high-dimensional data, dimensionality reduction is therefore typically performed beforehand (Macosko et al. 2015). In this study, the Mutual Nearest Neighbor (MNN) and Uniform Manifold Approximation and Projection (UMAP) algorithms were applied for dimensionality reduction, and UMAP was used to visualize the cell clusters. Clustering was optimized using the Shared Nearest Neighbor (SNN) algorithm to enhance cell grouping accuracy. Marker genes were identified in Seurat through differential expression testing using the bimod test, which allowed for the distinction of specific marker genes showing high expression in certain cell groups and low or no expression in others.

### Pseudotime trajectory analysis

Pseudotime trajectories of single-cell transcriptomic data were analyzed using Monocle 3 (Trapnell et al. 2014). The data were pre-processed using the estimateSizeFactors and estimatedispersion functions, followed by differential gene testing to order genes. Ordered genes were labeled with the setOrderingFilter function, and parameters were optimized to model potential developmental pathways.

### Differential gene and enrichment analysis

Differentially expressed genes were screened using the Metascape function in Seurat, with selection criteria of *p*-value < 0.05 and fold change > 1.5. Gene Ontology (GO) enrichment analysis of these differentially expressed genes was conducted on Metascape (www.metascape.org/).

### Construction of the transcriptional regulatory network

Initially, the Arabidopsis transcription factor database was retrieved from PlantTFDB (http://planttfdb.cbi.pku.edu.cn/). Using this resource, transcription factors were identified from the list of differentially expressed genes (DEGs) within each cell cluster. Subsequently, the PlantRegMap database (http://plantregmap.gao-lab.org/download.php) was accessed to obtain data on transcription factor interactions, enabling the determination of regulatory relationships between transcription factors and their target genes. By systematically organizing the interaction data between transcription factors and their corresponding target genes, a comprehensive regulatory relationship file was generated. Finally, Cytoscape (http://www.cytoscape.org/) was employed to visualize and construct the transcription factor regulatory network, providing a detailed representation of the intricate gene regulatory relationships.

### DNA extraction from plant tissues

Total genomic DNA was extracted following a modified CTAB protocol. Approximately 0.1 g of Arabidopsis tissue was ground to a fine powder under liquid nitrogen using a tissue homogenizer. The resulting powder was promptly mixed with 650 µL of preheated CTAB buffer (65 °C) and vortexed thoroughly. An equal volume of chloroform was added, gently mixed, and centrifuged at 12,000 rpm for 15 min at room temperature. The upper aqueous phase was carefully transferred to a fresh 1.5 mL EP tube, and DNA was precipitated by adding an equal volume of isopropanol. After incubation at room temperature for 10 min, the DNA solution was passed through a spin column and centrifuged at 12,000 rpm for 10 min, with the flow-through discarded. The column was washed twice with 70% ethanol, with each wash followed by centrifugation at 12,000 rpm for 1 min. To ensure complete ethanol evaporation, the column was centrifuged again at 12,000 rpm for 2 min. The column was then transferred to a new 1.5 mL tube, with the lid left open for a few minutes to allow residual ethanol to air-dry. Finally, 35–50 µL of preheated (65 °C) sterile ddH_2_O was applied directly to the membrane center for DNA elution. After a 2-min incubation at room temperature, DNA was collected by centrifugation at 12,000 rpm for 2 min. The eluted DNA samples were stored at −20 °C until further use.

### RNA extraction and reverse transcription

Total RNA was extracted using the TRIzol reagent kit (Invitrogen, ThermoFisher Scientific, Lenexa, KS), with all materials pre-treated with DEPC water (Merck, Sigma-Aldrich, St. Louis, MO) to eliminate RNase contamination. Approximately 0.1 g of plant tissue was ground in liquid nitrogen using a homogenizer, followed by the addition of 1 mL lysis buffer per sample, vigorous mixing, and a 5-min incubation at 15–30 °C. Samples were then centrifuged at 12,000 rpm for 10 min at 4 °C, and the Supernatant was transferred to RNase-free tubes. An equal volume of chloroform was added, mixed vigorously for 15 s, incubated at room temperature for 3 min, and centrifuged at 12,000 rpm for 10 min at 4 °C. The upper aqueous phase was mixed with an equal volume of ethanol and gently pipetted before transferring to an RNA spin column, followed by centrifugation at 12,000 rpm for 1 min. Subsequently, 500 µL protein removal buffer and 500 µL wash buffer were sequentially added to the column, each followed by 1-min centrifugation at 12,000 rpm, with the flow-through discarded after each wash. The column was centrifuged again at 12,000 rpm for 2 min to ensure removal of residual liquid. The RNA was eluted by applying 30–50 µL of RNase-free water to the membrane center, allowing a 2-min incubation at room temperature, followed by centrifugation at 12,000 rpm for 1 min. The eluted RNA was stored at −80 °C for future reverse transcription or downstream analyses.

### Synthesis of cDNA via RNA reverse transcription

cDNA was synthesized using the HiScript IV 1 st Strand cDNA Synthesis Kit (+ gDNA wiper) (Vazyme Biotech, Nanjing, China), following the manufacturer’s instructions. A 200 µL RNase-free microcentrifuge tube was used, to which 0.1 ng to 1 µg of total RNA and 1 µL of gDNA Purge were added, with RNase-free water added to adjust the final volume to 10 µL. The mixture was first incubated at 42 °C for 5 min to degrade residual genomic DNA, then placed on ice for 5 min. Next, 5 µL of 4 × HiScript IV RT SuperMi and 1 µL of Oligo (dT)20VN were added and gently mixed, after which the reaction was incubated at 50 °C for 15 min to complete reverse transcription. The reaction was terminated by heating at 85 °C for 5 min, and the synthesized cDNA was stored at −20 °C.

### qRT-PCR analysis for gene expression quantification

Gene expression levels were quantified through real-time PCR using SYBR Green I dye (Novoprotein Scientific Inc., Suzhou, China). Each PCR reaction contained 10 µL of 2 × Novo Start® SYBR qPCR Super Mix Plus, 1.5 µL of forward primer (10 µM), 1.5 µL of reverse primer (10 µM), 1 µL of cDNA template, and RNase-free water, adjusted to a final volume of 20 µL. The reaction mixture was transferred to a white 96-well plate, sealed, and briefly centrifuged to ensure homogeneity. Real-time PCR was performed on a qTOWER3G thermocycler with the following conditions: an initial denaturation at 95 °C for 1 min, followed by 40 cycles of 95 °C for 20 s, 60 °C for 20 s, and 72 °C for 30 s. Relative gene expression levels were calculated after amplification.

### Vector construction and transformation

The native promoter fragment of the target gene was amplified using genomic DNA from WT Arabidopsis as the template, employing KOD One™ PCR Master Mix (Merck, Sigma-Aldrich, St. Louis, MO). The PCR product was purified by gel extraction with FastPure Gel DNA Extraction Mini Kit (Vazyme Biotech, Nanjing, China) and cloned into the pCAMBIA1305.1 binary vector, replacing the 35S promoter upstream of the GUS reporter gene at KpnI and NcoI restriction sites using ClonExpress II One Step Cloning Kit (Vazyme Biotech, Nanjing, China).

The vector was Subjected to double digestion, and the resulting fragments were purified by gel extraction. The reaction mix for homologous recombination contained 2 µL Exnase II, 4 µL 5 × CE II Buffer, 0.04 ng of PCR product, and 0.04 ng of linearized vector, with ddH₂O added to a final volume of 20 µL. The mixture was incubated at 37 °C for 30 min and then cooled to 4 °C.

Chemically competent *Escherichia coli* (*E. coli*) cells (100 µL) were thawed on ice, and 10 µL of recombinant plasmid was added. The cells were gently mixed, incubated on ice for 45 min, heat-shocked at 42 °C for 90 s, and then returned to ice for 5 min. Next, 900 µL of LB medium without antibiotics was added, and the cells were incubated at 37 °C, 220 rpm for 1 h for recovery. After centrifugation at 5,000 rpm for 5 min, most of the Supernatant was discarded, and the pellet was resuspended in 30–50 µL of LB, spread onto selective LB agar plates with kanamycin, and incubated overnight at 37 °C. Positive colonies were selected and confirmed by colony PCR.

Positive *E. coli* clones were cultured in LB medium containing kanamycin and confirmed by PCR. Plasmid DNA was extracted from PCR-confirmed clones and sequenced to confirm the correct insertion of the target sequence. Plasmids from correctly sequenced clones were extracted for further use.

Competent *Agrobacterium tumefaciens* (*Agrobacterium*) cells were thawed on ice, and 1 µg of plasmid DNA was added to 100 µL of cells. The mixture underwent freeze–thaw cycling as follows: 5 min on ice, 5 min in liquid nitrogen, 5 min at 37 °C, and then returned to ice. Subsequently, 800 µL of antibiotic-free YEP medium was added, and the cells were recovered at 28 °C, 200 rpm for 2–3 h. After centrifugation at 5,000 rpm for 5 min, the pellet was resuspended in approximately 50 µL of the medium and spread on LB agar plates with kanamycin and rifampicin. Plates were incubated at 37 °C for 2–3 days to observe positive colonies. Positive *Agrobacterium* colonies were cultured and confirmed by PCR before further use.

### Generation of transgenic Arabidopsis lines

Four-week-old WT Arabidopsis plants with unopened flower buds were transformed through *Agrobacterium*-mediated floral dipping. *Agrobacterium* colonies were initially cultivated on YEP solid medium containing antibiotics, then inoculated into 3–5 mL YEP liquid medium and incubated overnight at 28 °C with shaking at 200 rpm. The next day, the culture was expanded to 100 mL of YEP medium and grown to an OD600 of 1.2–1.6. Cells were harvested by centrifugation and resuspended in infiltration solution (1/2 MS medium with 5% sucrose) to reach an OD600 of 0.6–0.8. Arabidopsis inflorescences were dipped in the Suspension for 20 s, incubated in darkness for 24 h, and subsequently cultivated to maturity under standard conditions. Seeds were then harvested for selection. Antibiotic-resistant seedlings were transferred to soil for propagation, and the subsequent generation was screened by PCR to identify positive transgenics. Stable T3 homozygous lines were selected for further analysis.

### GUS staining

A 20 mM stock solution of X-Gluc was prepared in N, N-Dimethylformamide (DMF) and diluted 1:9 to create the GUS staining solution, which was stored at −20 °C in darkness. Samples were immersed in the staining solution and incubated at 37 °C in darkness for 1–24 h until progressive blue staining developed in the tissues. After staining, samples were transferred to 70% ethanol for decolorization until clear. The GUS expression pattern was observed with a stereomicroscope to identify blue staining sites.

### Environmental Scanning Electron Microscopy (ESEM) observation

Callus tissues at various developmental stages were excised, labeled, and fixed in 2.5% glutaraldehyde for imaging, then stored under vacuum overnight, followed by three washes with 0.1 M PBS for 15 min each. Dehydration was performed through a graded ethanol series (30%–100%), followed by immersion in pure tert-butanol twice. Samples were subsequently frozen at −20 °C to crystallize tert-butanol, freeze-dried, and sputter-coated with gold using a magnetron sputter coater.

## Supplementary Information

Supplementary Material 1. Fig. S1 Quality control after data quantification. Fig. S2 GO and KEGG analysis of DEGs in each cell cluster of different samples. Fig. S3 Tissue expression patterns of selected marker genes. Fig. S4 Distribution analysis of different clusters in the pseudo-temporal trajectory of leaf-derived callus. Fig. S5 Distribution analysis of different clusters in the pseudo-temporal trajectory of root-derived callus. Fig. S6 Statistical analysis of fresh weight of callus tissue from WT, wrky33 and WRKY33-MYC. Fig. S7 Developmental progression of leaf and root callus under hypoxic stress conditions. Fig. S8 Effects of the prt6 mutant on callus development. Fig. S9 Effect of the constitutive photomorphogenic signaling mutant cop1 on leaf and root callus development. Fig. S10 Effect of the mutant hy5 on leaf and root callus development. Fig. S11 qRT-PCR analysis of LHCB, PTL, and WOX5 gene expression at different developmental stages in WT and hy5 mutants. Fig. S12 Analysis of the impact of salt stress on callus development. Fig. S13 Analysis of the impact of the salt stress response-deficient mutant rrtf1 on callus development

Supplementary Material 2. Table S1. The DEGs for each cluster of leaf sample. Table S2. The GO analysis of the DEGs for each cluster of leaf sample. Table S3. The DEGs for each cluster of root sample. Table S4. The GO analysis of the DEGs for each cluster of root sample. Table S5. The DEGs for each cluster of integrated leaf and root sample. Table S6. The DEGs of leaf_callus-vs-leaf_initial_callus. Table S7. The DEGs of leaf_callus_greening-vs-leaf_callus. Table S8. The genes list in profiles of leaf sample. Table S9. The DEGs of root_callus-vs-root_initial_callus. Table S10. The DEGs of root_callus_greening-vs-root_callus. Table S11. The genes list in profiles of root sample. Table S12. Primers for constructing GUS reporter gene transformation vectors. Table S13. Primers for qRT-PCR

## Data Availability

The raw sequence data from the single-cell RNA sequencing (scRNA-seq) conducted in this study have been deposited in the National Center for Biotechnology Information (NCBI) and are accessible at the following web address: https://www.ncbi.nlm.nih.gov/bioproject/803816 under accession number SRA: SRS12398586.

## Abbreviations

SEM: Scanning electron microscopy
QC: Quality control
UMIs: Unique molecular identifiers
UMAP: Uniform Manifold Approximation and Projection
DEGs: Differentially expressed genes
GO: Gene Ontology
scRNA-seq: Single-cell RNA sequencing
qRT-PCR: Quantitative reverse transcription polymerase chain reaction
KEGG: Kyoto Encyclopedia of Genes and Genomes
PAGA: Partition-based graph abstraction
ABA: Abscisic acid
STEM: Short Time-Series Expression Miner
