# Supplementary material for: Single-cell RNA sequencing reveals developmental trajectories and environmental regulation of callus formation in Arabidopsis
Source: Stress Biol. 2025 Sep 12;5(1):57. doi: 10.1007/s44154-025-00255-4 (PMC12431987; doi:10.1007/s44154-025-00255-4)
Supplement: Supplementary file 1 — Supplementary Material 1. Fig. S1 Quality control after data quantification. Fig. S2 GO and KEGG analysis of DEGs in each cell cluster of different samples. Fig. S3 Tissue expression patterns of selected marker genes. Fig. S4 Distribution analysis of different clusters in the pseudo-temporal trajectory of leaf-derived callus. Fig. S5 Distribution analysis of different clusters in the pseudo-temporal trajectory of root-derived callus. Fig. S6 Statistical analysis of fresh weight of callus tissue from WT, wrky33 and WRKY33-MYC. Fig. S7 Developmental progression of leaf and root callus under hypoxic stress conditions. Fig. S8 Effects of the prt6 mutant on callus development. Fig. S9 Effect of the constitutive photomorphogenic signaling mutant cop1 on leaf and root callus development. Fig. S10 Effect of the mutant hy5 on leaf and root callus development. Fig. S11 qRT-PCR analysis of LHCB, PTL, and WOX5 gene expression at different developmental stages in WT and hy5 mutants. Fig. S12 Analysis of the impact of salt stress on callus development. Fig. S13 Analysis of the impact of the salt stress response-deficient mutant rrtf1 on callus development [file 44154_2025_255_MOESM1_ESM.docx]

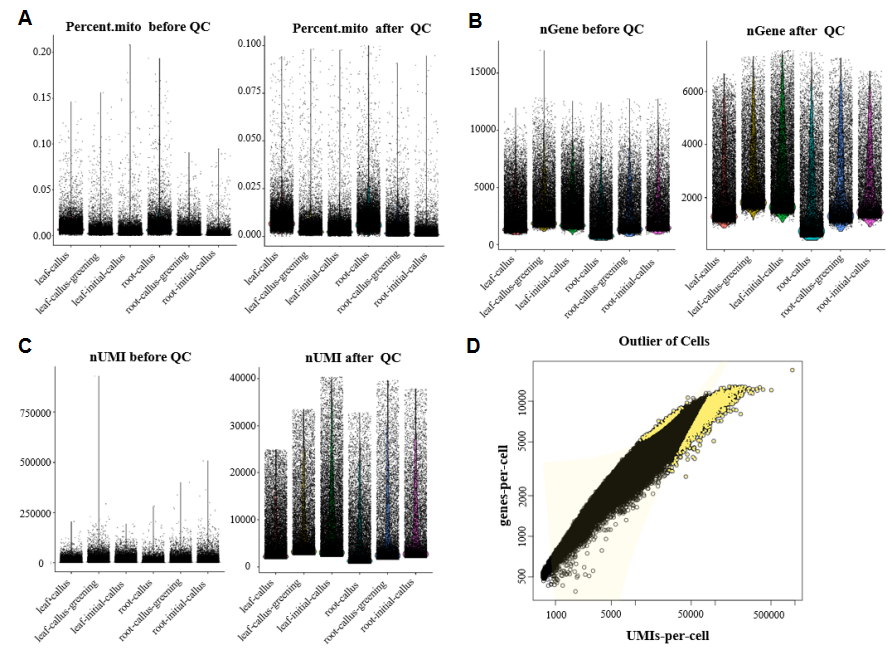


**Fig. S1** Quality control after data quantification.

Filter out data with double cells, multiple cells, or unbound cells through data quality control. **A** Violin distribution map of mitochondrial gene proportion in each cell before and after quality control. **B** Violin distribution map of gene expression numbers in each cell before and after quality control. **C** Violin distribution map of UMI number in each cell before and after quality control. **D** Fit a generalized linear model to remove delocalized cells, and fit a distribution model based on the linear relationship between the two. The colored points represent delocalized cells, which will be removed in downstream analysis.


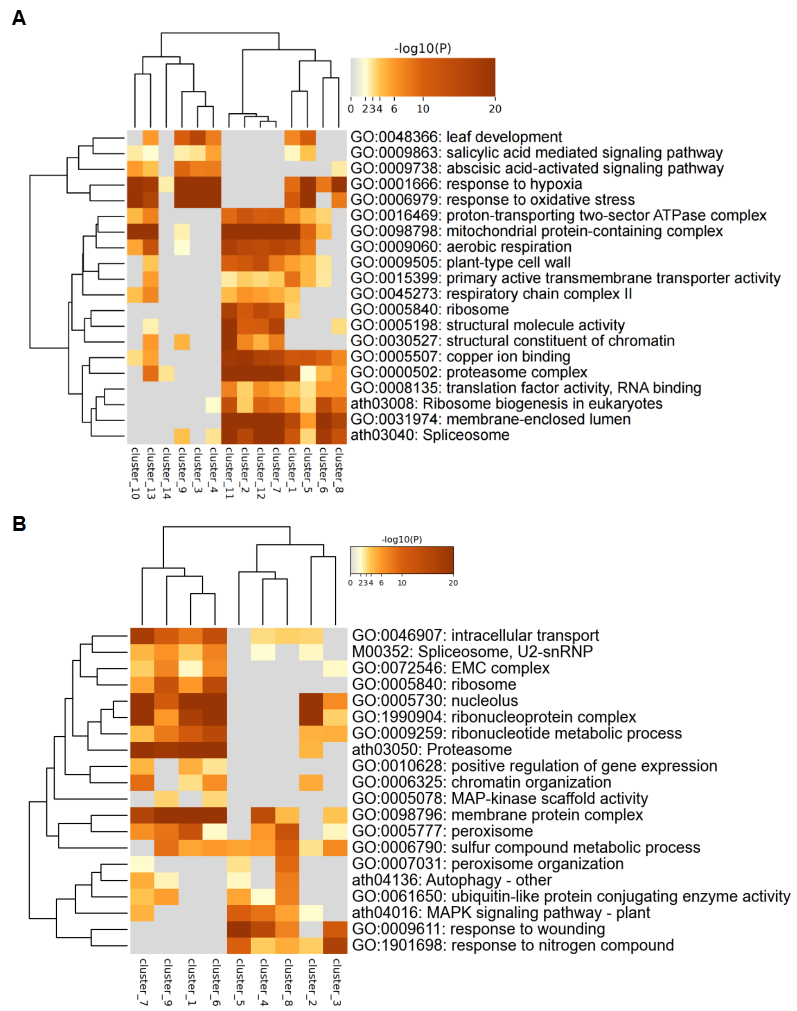


**Fig. S2** Gene Ontology (GO) and Kyoto Encyclopedia of Genes and Genomes (KEGG) analysis of DEGs in each cell cluster of different samples.

**A** GO heatmap showing the enrichment of GO terms in each of cell clusters of leaf-derived callus. **B** GO heatmap showing the enrichment of GO terms in each of cell clusters of root-derived callus.


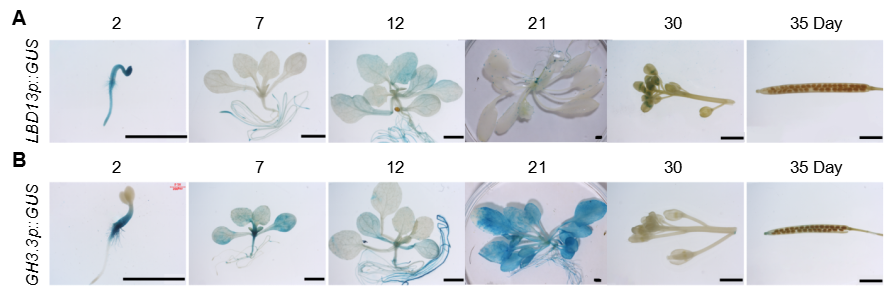


**Fig. S3** Tissue expression patterns of selected marker genes.

**A** Expression patterns of *LBD13p::GUS* at different developmental stages and in various tissues of *Arabidopsis*. (Scale bar: 2 mm)

**B** Expression patterns of *GH3.3p::GUS* at different developmental stages and in various tissues of *Arabidopsis*. (Scale bar: 2 mm)


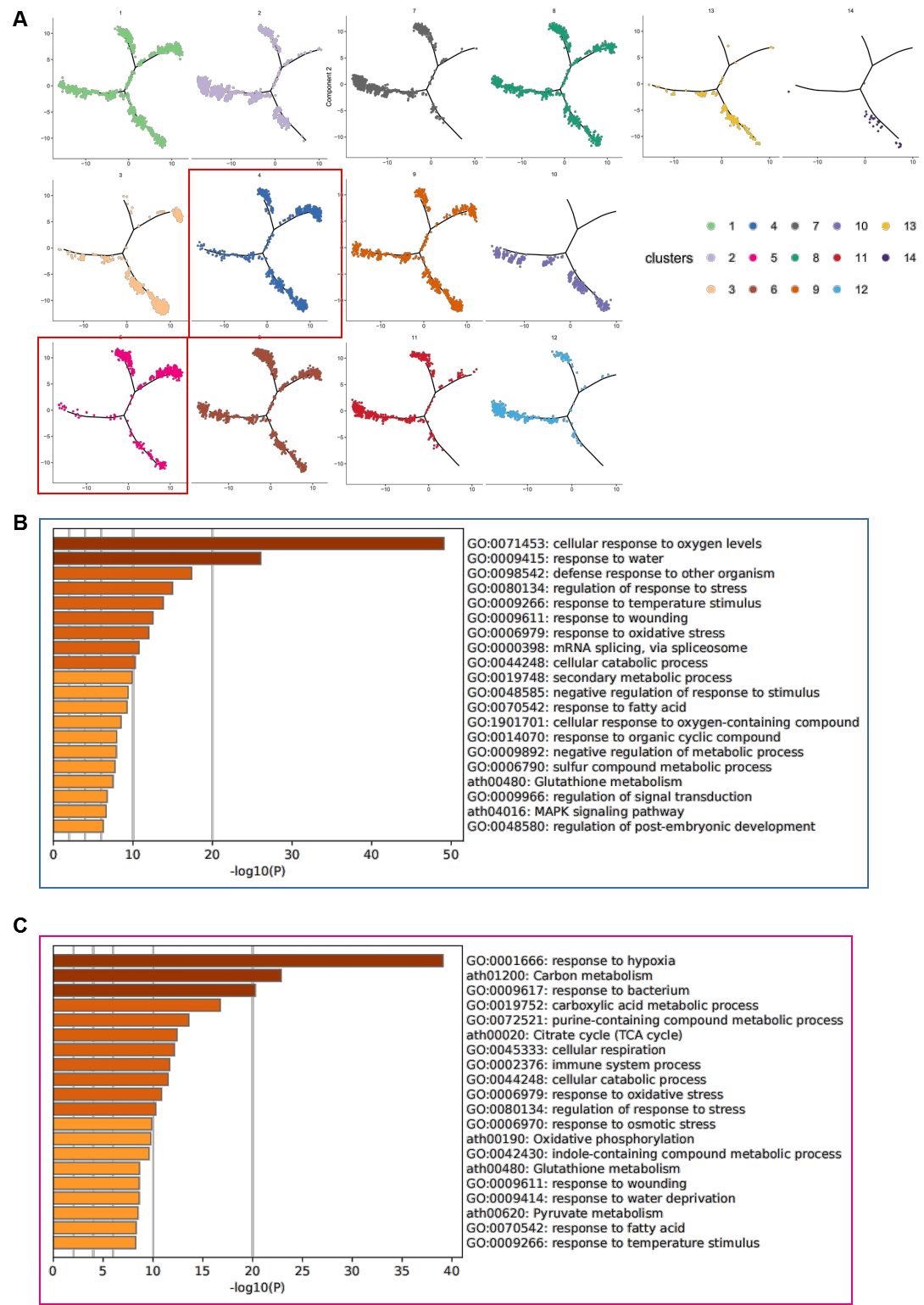


**Fig. S4** Distribution analysis of different clusters in the pseudo-temporal trajectory of leaf-derived callus. **A** The cell_trajectory plot depicting the distribution of leaf-derived callus cells along the pseudo-temporal trajectory, with cells grouped according to each cluster. **B** GO and KEGG pathway analyses of genes specifically expressed in Cluster 4. **C** GO and KEGG pathway analyses of genes specifically expressed in Cluster 5.


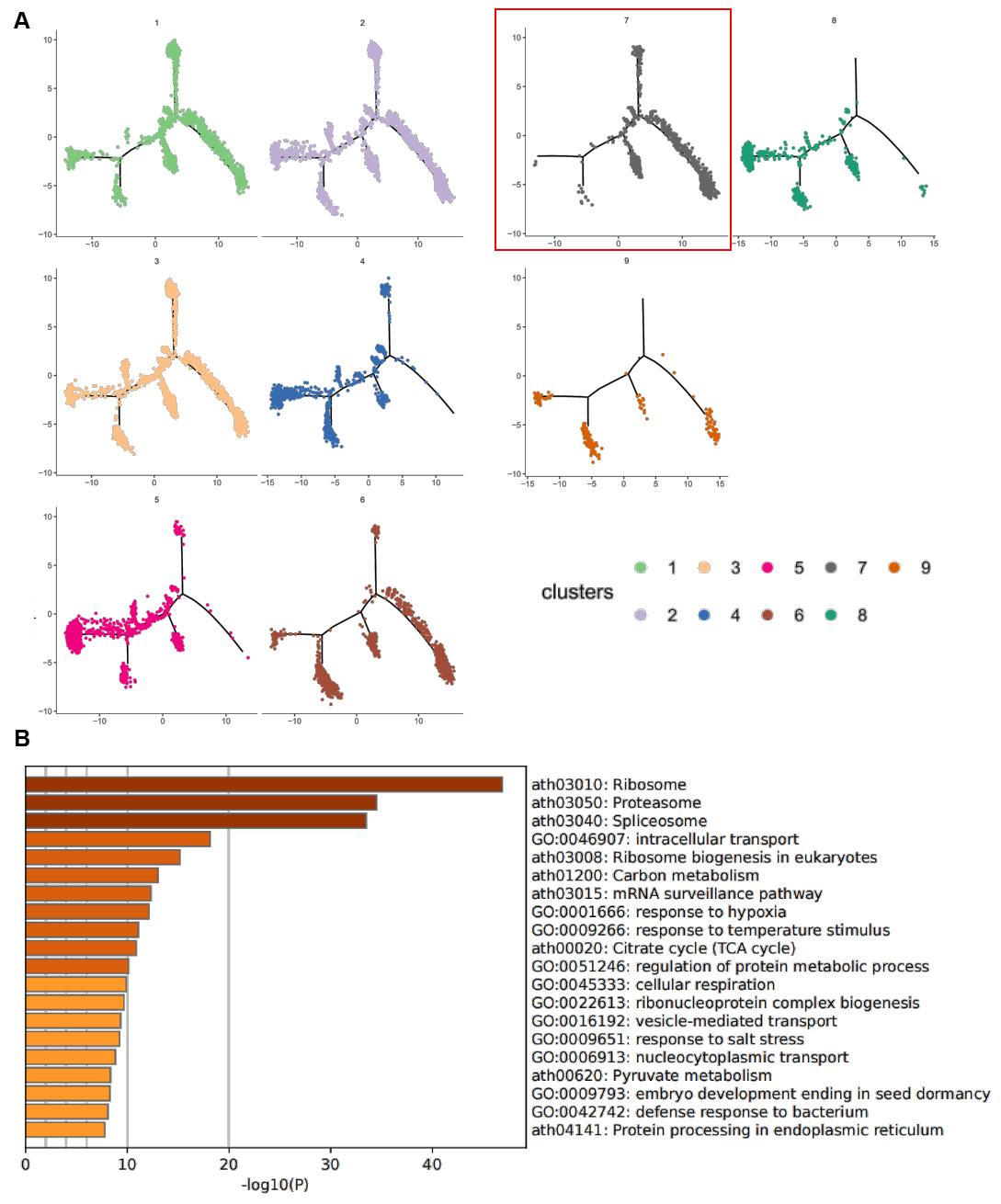


**Fig. S5** Distribution analysis of different clusters in the pseudo-temporal trajectory of root-derived callus. **A** The cell_trajectory plot depicting the distribution of root-derived callus cells along the pseudo-temporal trajectory, with cells grouped according to each cluster. **B** GO and KEGG pathway analyses of genes specifically expressed in Cluster 7.

**Fig. S6** Statistical analysis of fresh weight of callus tissue from WT, *wrky33* and *WRKY33-MYC*.

Quantitative analysis of fresh weight for leaf callus under both treatment conditions.


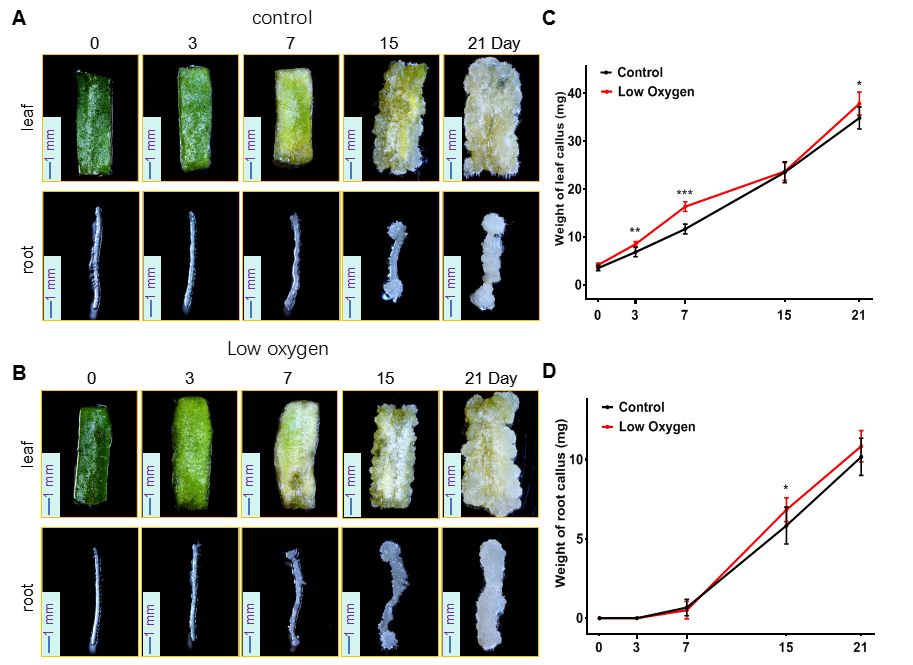


**Fig. S7** Developmental progression of leaf and root callus under hypoxic stress conditions.

**A** Phenotypic analysis of WT *Arabidopsis* leaf and root callus under normal oxygen conditions (7% oxygen concentration, scale bar = 1 mm).

**B** Phenotypic analysis of WT *Arabidopsis* leaf and root callus under low oxygen conditions (3% oxygen concentration, scale bar = 1 mm).

**C** Quantitative analysis of fresh weight for leaf callus under both treatment conditions.

**D** Quantitative analysis of fresh weight for root callus under both treatment conditions. A Student's t-test was used to assess statistical significance between low oxygen and normal conditions (n=3). Error bars represent standard deviation. Significance levels: *** p<0.001, **p<0.01, and * p<0.05.


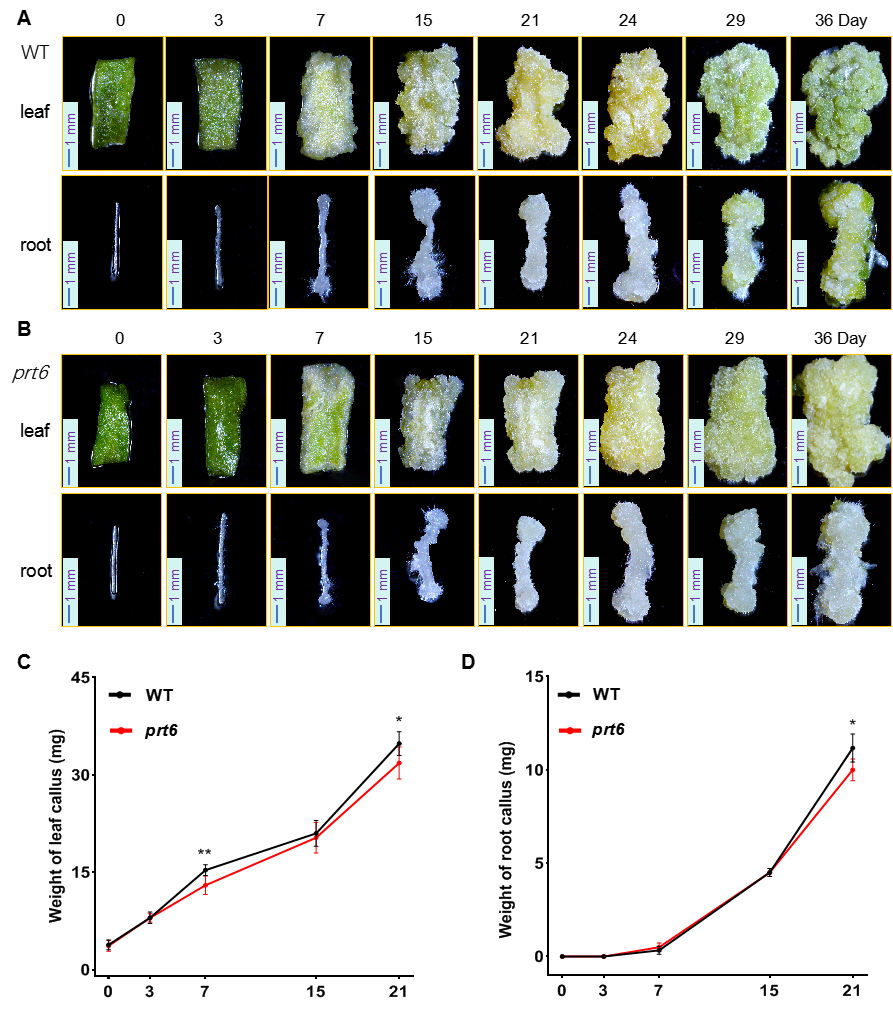


**Fig. S8** Effects of the *prt6* mutant on callus development.

**A** Growth phenotypes of WT leaf and root callus (scale bar = 1 mm).

**B** Growth phenotypes of *prt6* mutant leaf and root callus (scale bar = 1 mm).

**C** Quantitative analysis of fresh weight for WT and *prt6* mutant leaf callus.

**D** Quantitative analysis of fresh weight for WT and *prt6* mutant root callus. A Student's t-test was performed to evaluate significant differences between WT and *prt6* mutant (n=6). Error bars represent standard deviation. Significance levels: ** p<0.01, and * p<0.05.


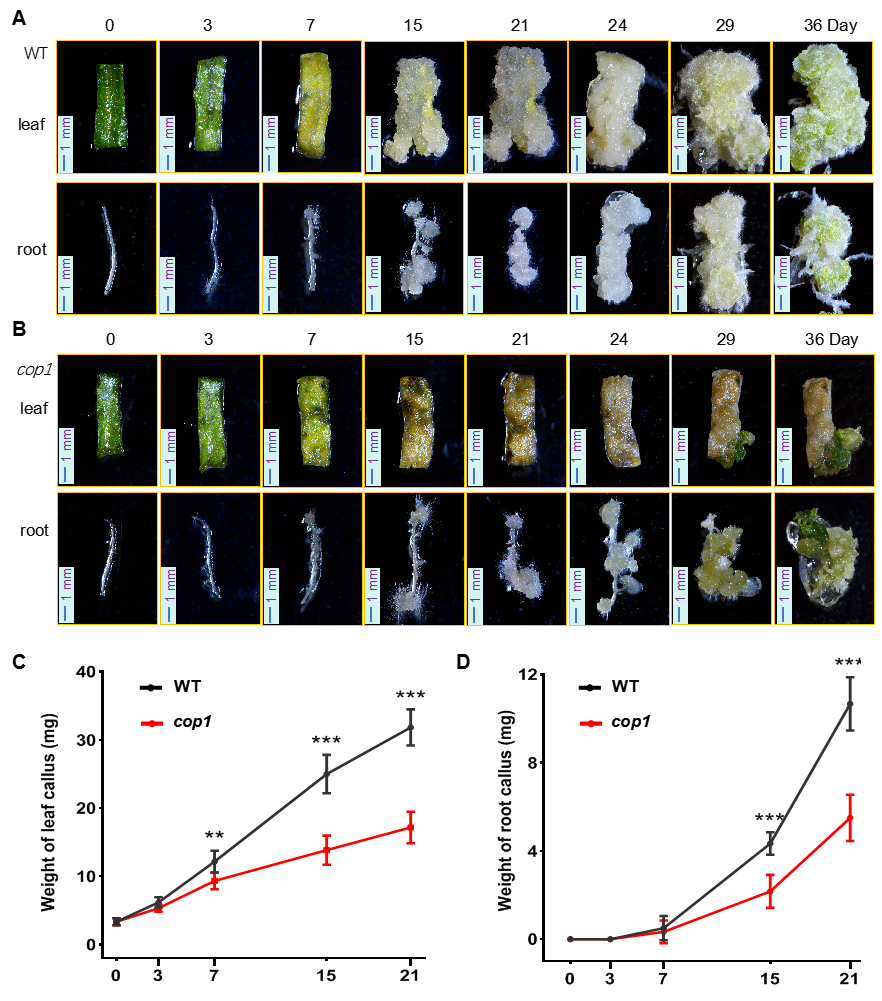


**Fig. S9** Effect of the constitutive photomorphogenic signaling mutant *cop1* on leaf and root callus development.

**A** Growth phenotypes of wild-type (WT) leaf and root callus (scale bar = 1 mm).

**B** Growth phenotypes of *cop1* mutant leaf and root callus (scale bar = 1 mm).

**C** Statistical analysis of fresh weight of leaf callus in WT and *cop1* mutant.

**D** Statistical analysis of fresh weight of root callus in WT and *cop1* mutant.

Significant differences between *cop1* mutants and WT were determined using Student’s t-test (n=6). Error bars represent standard deviation (SD), with significance levels indicated as ***p < 0.001, **p < 0.01, and *p < 0.05.


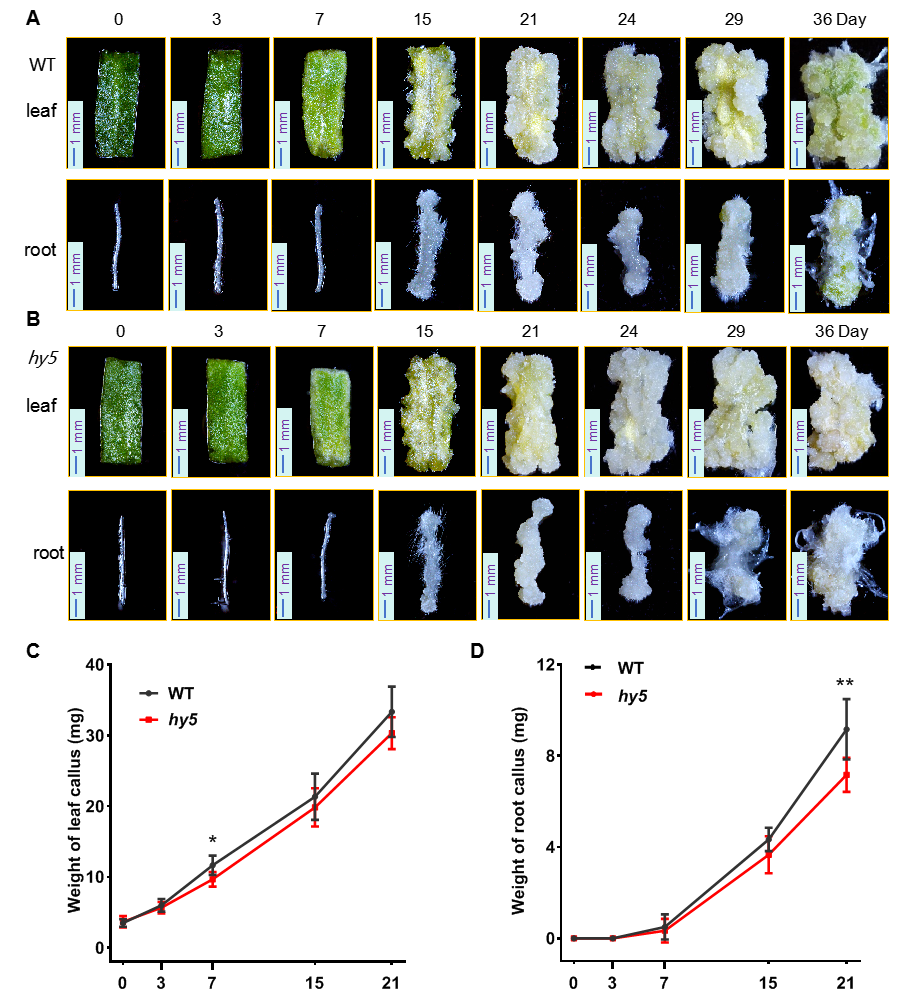


**Fig. S10** Effect of the mutant *hy5* on leaf and root callus development.

**A** Growth phenotypes of wild-type (WT) leaf and root callus (scale bar = 1 mm).

**B** Growth phenotypes of *hy5* mutant leaf and root callus (scale bar = 1 mm).

**C** Statistical analysis of fresh weight of leaf callus in WT and *hy5* mutant.

**D** Statistical analysis of fresh weight of root callus in WT and *hy5* mutant.

Statistical significance between *hy5* mutants and WT was evaluated using Student’s t-test (n=6). Error bars indicate SD, with significance levels shown as **p < 0.01, and *p < 0.05.


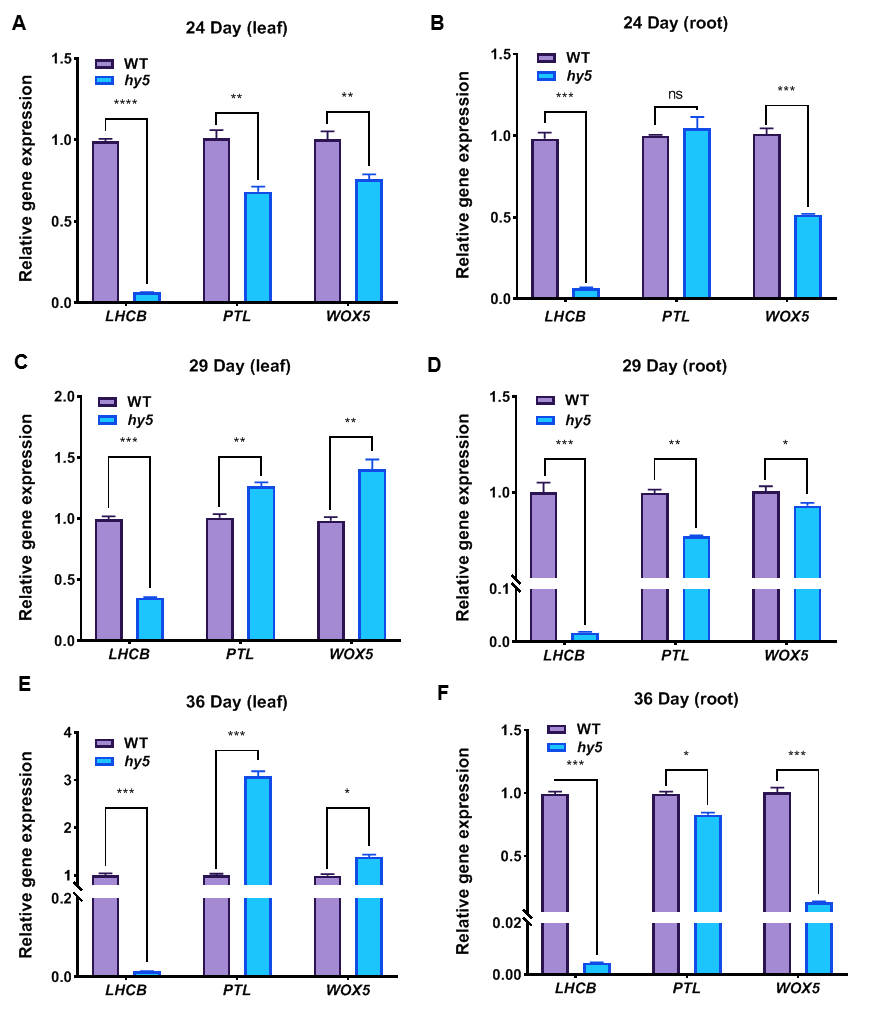


**Fig. S11** qRT-PCR analysis of *LHCB*, *PTL*, and *WOX5* gene expression at different developmental stages in WT and *hy5* mutants.

(**A**, **C**, **E**) qRT-PCR analysis of *LHCB*, *PTL*, and *WOX5* gene expression in leaf callus from WT and *hy5* mutants at 24, 29, and 36 days post-treatment.

(**B**, **D**, **F**) qRT-PCR analysis of *LHCB*, *PTL*, and *WOX5* gene expression in root callus from WT and *hy5* mutants at 24, 29, and 36 days post-treatment.

*ACTIN2* was used as the internal control. WT callus at corresponding stages was used as the reference. Student’s t-test was applied to assess significant differences between *hy5* mutants and WT (n=3). Error bars represent SD, with significance denoted as ***p < 0.001, **p < 0.01, and *p < 0.05.


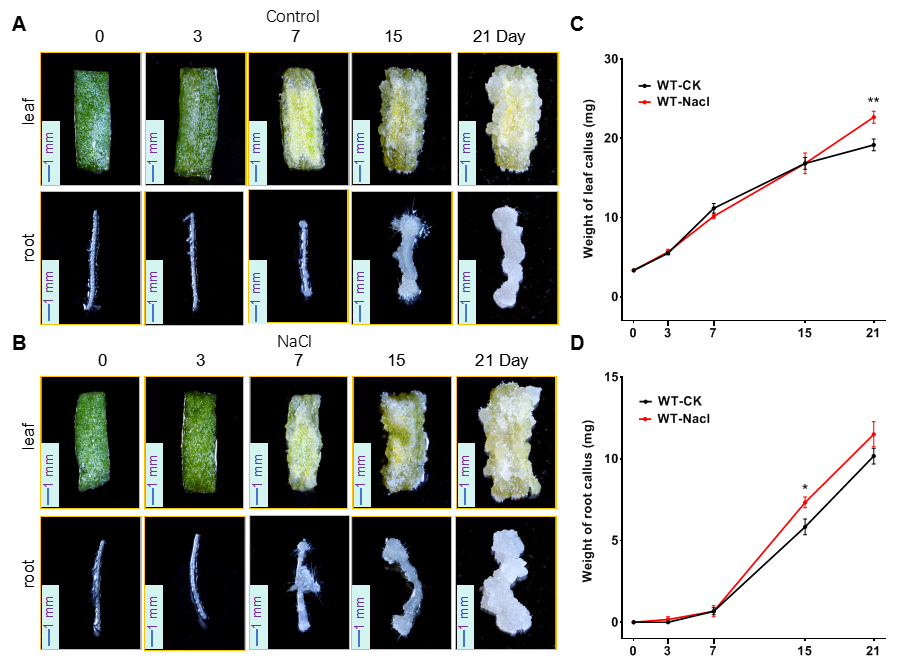


**Fig. S12** Analysis of the impact of salt stress on callus development.

**A** Phenotypic observations of leaf and root callus growth in wild-type (WT) *Arabidopsis* under normal cultivation conditions (scale bar = 1 mm).

**B** Phenotypic observations of leaf and root callus growth in *Arabidopsis* subjected to 50 mM NaCl supplementation (scale bar = 1 mm).

**C** Weight measurements of leaf callus from both experimental groups.

**D** Weight measurements of root callus from both experimental groups. A Student's t-test was performed to assess significant differences between NaCl-stressed and control conditions (n = 6), with error bars denoting standard deviation (SD). Significance levels are indicated as **p < 0.01, and *p < 0.05.


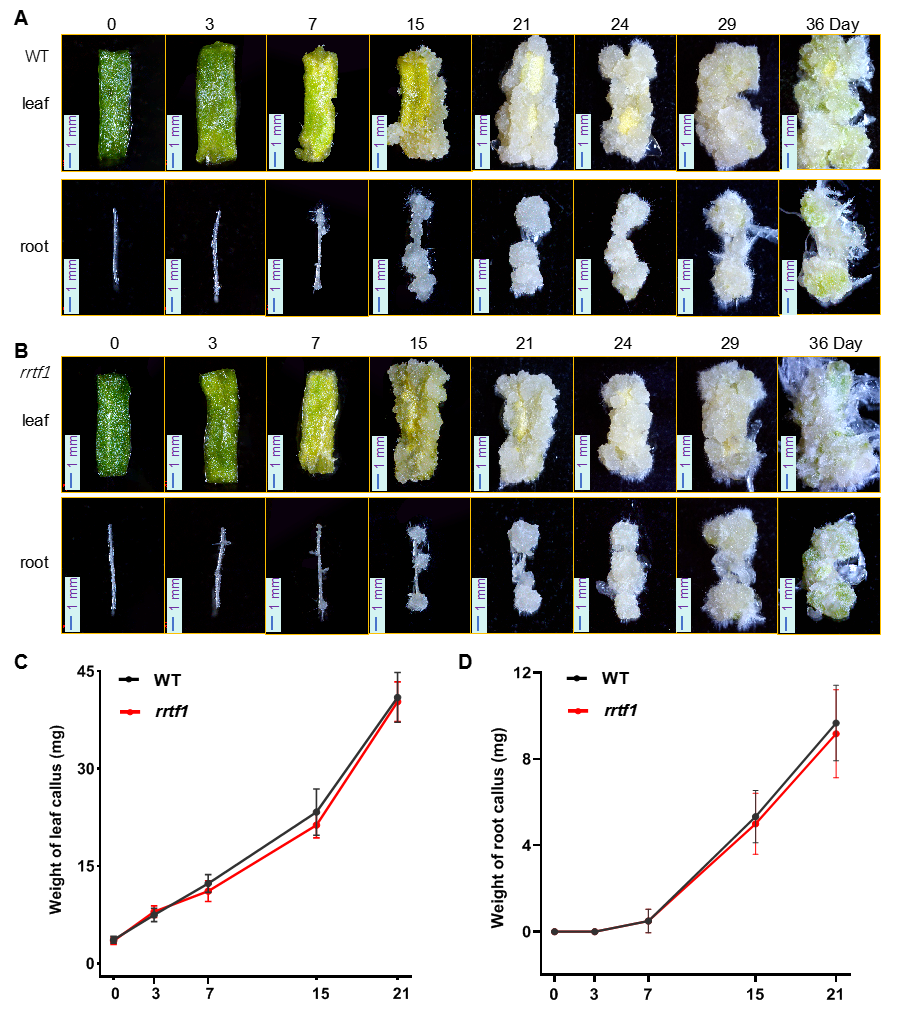


**Fig. S13** Analysis of the impact of the salt stress response-deficient mutant *rrtf1* on callus development.

**A** Phenotypic observations of leaf and root callus growth in wild-type (WT) *Arabidopsis* (scale bar = 1 mm).

**B** Phenotypic observations of leaf and root callus growth in the *rrtf1* mutant (scale bar = 1 mm).

**C** Weight measurements of leaf callus from WT and *rrtf1* mutants.

**D** Weight measurements of root callus from WT and *rrtf1* mutants. A Student's t-test was utilized to evaluate significant differences between the *rrtf1* mutant and WT (n = 6), with error bars representing standard deviation (SD).
